# Isohemagglutinins exhibit synergistic polyreactivity toward *Streptococcus pneumoniae* surface antigens: implications for broad-spectrum reactivity of human antibodies

**DOI:** 10.1007/s00430-025-00862-y

**Published:** 2026-01-09

**Authors:** Jens Magnus Bernth Jensen, Ole Schmeltz Søgaard, Mikkel Steen Petersen, Steen Hoffmann, Bjarne Kuno Møller, Annette Gudmann Hansen, Uffe B. Skov Sørensen, Steffen Thiel

**Affiliations:** 1 Department of Clinical Immunology, Aarhus University Hospital, Aarhus, Denmark; 2 Department of Molecular Medicine, Aarhus University Hospital, Aarhus, Denmark; 3 Department of Infectious Diseases, Aarhus University Hospital, Aarhus, Denmark; 4 Department of Bacteria, Parasites and Fungi, Statens Serum Institut, Copenhagen, Denmark; 5 Department of Biomedicine, Aarhus University, Aarhus, Denmark

**Keywords:** Streptococcus pneumoniae, ABO blood group antigens, Isohemagglutinins, Naturally occurring antibodies, Antigen–antibody interactions, Synergistic polyreactivity

## Abstract

Synergistic polyreactivity of antibodies—where distinct oligo- or polyreactive clones combine to enable broad-spectrum antigen binding—was recently demonstrated for human anti-galactose-α-1,3-galactose (αGal) antibodies. However, it remains unclear whether this phenomenon also occurs in other antigen-defined antibody populations. Here, we investigated synergistic polyreactivity in isohemagglutinins (naturally occurring antibodies against ABO blood group antigens) and its potential role in antimicrobial defense. Isohemagglutinins were affinity-purified from pooled plasma from groups of healthy donors sharing the same ABO blood group, then characterized by solid-phase immunoassays, SDS-PAGE, and Western blotting. Binding to formaldehyde-fixed *Streptococcus pneumoniae* representing 30 serotypes was assessed by flow cytometry. Specificity was further examined using inhibition assays with soluble pneumococcal polysaccharides. To evaluate clinical relevance, ABO blood group types (used as proxies for isohemagglutinin profiles) were linked to serotype-specific invasive pneumococcal disease (IPD) events using Danish nationwide registry data. The purified isohemagglutinins retained specificity for their cognate ABO antigens. Notably, they bound all tested pneumococcal strains, primarily through reactivity with the common cell wall polysaccharide. Adsorption studies further revealed serotype-specific capsule binding mediated by distinct isohemagglutinin clones, demonstrating synergistic polyreactivity. Despite this, population-level analyses showed only modest protection: most notably, individuals with blood types B or O (producers of anti-A and anti-A,B isohemagglutinins, respectively) had a 19% lower prevalence of serotype 9V IPD than blood type A or AB individuals. Although the overall protective effect of isohemagglutinins against IPD seems limited, the observed synergistic polyreactivity supports the concept that this is a general feature of antigen-defined antibodies, extending beyond anti-αGal antibodies.

**Supplementary Information:**

The online version contains supplementary material available at 10.1007/s00430-025-00862-y.

## Introduction

Antibodies were historically thought to be strictly monospecific, meaning that each antibody was believed to bind exclusively to a single, specific epitope, as described by the classical ‘lock-and-key’ model of antigen recognition [1]. However, subsequent research has established that antibodies can exhibit polyreactivity, allowing a single antibody to bind multiple structurally distinct epitopes [2]. Polyreactive antibodies are now recognized as a substantial component of the human antibody repertoire, playing key roles in immune defense against pathogens [3, 4]. Despite this recognition, their significance is largely extrapolated from studies on engineered monoclonal antibodies [5–8], with limited investigation into naturally occurring polyreactive antibodies in humans. 

*Streptococcus pneumoniae* offers an attractive model for studying antibody polyreactivity due to the structural diversity of its capsular polysaccharides. These chemically distinct, surface-exposed polysaccharides define serotype specificity, with approximately 100 known serotypes, most of which are well characterized at the molecular level [9, 10]. This antigenic diversity provides a well-defined landscape for evaluating patterns of antibody reactivity. Moreover, encapsulated *S. pneumoniae* is a clinically significant pathogen, and antibodies targeting its capsule confer critical protection against pneumococcal infections [11, 12]. Thus, *S. pneumoniae* represents an immunologically relevant system for exploring the principles of human antibody reactivity to polysaccharide antigens.

Recently, we found that human plasma antibodies against terminal galactose-α-1,3-galactose (anti-αGal antibodies), which are considered the most abundant naturally occurring antibody in human plasma [13], exhibited reactivity to 48 out of 91 tested serotypes of *S. pneumoniae* [14]. The targeted serotypes encompassed a diverse range of carbohydrate structures, none of which contained terminal galactose-α-1,3-galactose. This broad-spectrum polyreactivity resulted from the synergistic action of multiple distinct antibodies within the anti-αGal antibody pool, each binding a different subset of tested carbohydrate structures in addition to their shared reactivity to galactose-α-1,3-galactose; suggestive of distinct oligo- or polyreactive capabilities across these antibodies. However, it remains unclear whether such synergistic polyreactivity is unique to human anti-αGal antibodies or represents a general feature of antibodies.

Isohemagglutinins represent another well-characterized group of naturally occurring human antibodies defined by antigen reactivity. These antibodies bind the human ABO blood group antigens A and B (terminal GalNAcα3[Fucα2]Galβ4GlcNAc-R and terminal Galα3[Fucα2]Galβ4GlcNAc-R, respectively) and circulate as IgM, IgG, and IgA classes in plasma [15]. Human populations exhibit four distinct ABO blood types, with their distribution influenced by ethnic background [16, 17]. Type A individuals express only the blood group A antigen, type B individuals express only the blood group B antigen, type AB individuals express both, and type O individuals express neither of the two antigens. In individuals lacking one or both antigens, the immune system produces isohemagglutinins against the absent antigen(s) [18, 19]. The mechanisms governing the production of these isohemagglutinins remain incompletely understood. Proposed factors include continuous immunization by A- and B-like substances from the gut microbiota, supported by reports that more than half of examined gram-negative gut bacteria bind isohemagglutinins [20, 21], and tolerance-mediated suppression of antibody production in individuals expressing the cognate antigens, consistent with established immunological principles [22]. Isohemagglutinins can initiate complement activation and phagocytosis upon binding to ABO antigens on cells, granting them a crucial clinical role in transfusion medicine and organ transplantation. However, their potential for synergistic polyreactivity—and its potential role in combating microorganisms—has not been explored.

In this study, we used isohemagglutinins as a model to investigate whether synergistic polyreactivity is a broader feature of antigen-defined antibodies. Furthermore, we aimed to assess the potential role of isohemagglutinin polyreactivity in protecting humans against pneumococcal infections.

## Methods

### Human blood derivatives

Surplus blood from healthy blood donor donations was obtained from the Blood Bank of the Department of Clinical Immunology, Aarhus University Hospital, Denmark.

### Plasma pools for affinity isolation of isohemagglutinins

Whole blood from healthy blood donors was collected in tubes containing ethylenediaminetetraacetic acid (EDTA) and centrifuged at 2,000 × g for 10 min to separate plasma. Four 100 mL plasma pools were prepared, each containing 1 mL of plasma from each of 100 different donors. One pool consisted of plasma from ABO type A donors, containing anti-B isohemagglutinins; a second pool consisted of plasma from ABO type B donors, containing anti-A isohemagglutinins; the two remaining pools contained plasma from ABO type O donors, who produce both anti-A and anti-B isohemagglutinins. These were used separately for the isolation of anti-A and anti-B isohemagglutinins, respectively.

Each 100 mL plasma pool was diluted with 400 mL Tris-buffered saline (TBS; 10 mM TRIS, 140 mM NaCl, 0.1% (v/v) NaN_3_, pH 7.4) containing Tween-20 (TBS/Tw, 0.05% (v/v) Tween-20) to a final volume of 500 mL. The diluted plasma was centrifuged at 10,000 × g for 10 min, and the resulting supernatants were filtered through gauze to produce the starting materials for isohemagglutinin affinity purification.

### Preparation of fixed Red Blood Cells (RBCs)

EDTA-stabilized donor blood of ABO type A or B was pooled separately for each blood type, with each pool containing 1 mL of blood from ten individuals. The following procedures were performed at ambient temperature, with centrifugation at 200 × g for 5 min between each step.

1. Washing: Pooled blood (10 mL) was diluted with 40 mL phosphate-buffered saline (PBS, pH 7.4) and washed twice by centrifugation.
2. Fixation: The cell pellets were resuspended in 50 mL PBS containing 2% (w/v) glucose and 0.25% (v/v) glutaraldehyde, followed by end-over-end rotation for 30 min.
3. Quenching: After centrifugation, the cells were resuspended in 50 mL PBS, then pelleted and resuspended in 50 mL PBS containing 50 mM ethanolamine, followed by end-over-end rotation for 15 min.
4. Final washes: The cells were pelleted by centrifugation and washed twice in 50 mL TBS.
5. Resuspension: The final cell suspension was adjusted to 5 mL in TBS with human serum albumin (TBS/HSA, 1 g/L).

The hematocrit, i.e., the percentage of the sample volume occupied by erythrocytes, was measured using a Sysmex XT-1800i (Sysmex Corporation, Japan).

### Affinity purification of isohemagglutinins

The starting materials (diluted plasma pools) were processed using columns containing 20 mL of beads conjugated with the blood group A antigen (A column) or blood group B antigen (B column) (Glycosorb^®^-ABO, Glycorex Transplantation AB, Sweden). The conjugated ABO antigens included a mixture of tri-, tetra-, and longer saccharides. The purification was performed at ambient temperature with a flow rate of approximately 40 mL per hour.

After loading the plasma, the columns were washed with 100 mL TBS/Tw, followed by an additional 200 mL TBS to remove unbound components. Isohemagglutinins were eluted using 0.1 M glycine buffer (pH 2.5) in 1 mL fractions. Eluates were collected into Tween-blocked tubes containing 85 µL of 1 M Tris-HCl buffer (pH 8.5) to neutralize the pH in the sample.

For each purification run, the following samples were collected and stored: the starting material, the entire flow-through (identified by its yellowish color, in 10 batches of 50 mL), the first 100 mL of the wash-out (in two 50-mL batches), and 40 sequential elution fractions.

### TRIFMAs (Time-Resolved ImmunoFluorometric Assays; solid-phase immunoassays)

The following antigens were used for coating of plates: HSA (CSL Behring, King of Prussia, PA, USA), blood group A antigen conjugate (Blood group A-HSA, NGP9305, Dextra Laboratories, Reading, UK), blood group B antigen conjugate (Blood group B-HSA, NGP9323, Dextra Laboratories), mannan (prepared as described by Nakajima and Ballou [23]; selected as a high-mannose structure to control for contaminating anti-polysaccharide antibodies), and tetanus toxoid (TT, product number 2674, Statens Serum Institut, Copenhagen, Denmark; selected as a control for contaminating anti-protein antibodies; anti-TT antibodies are anticipated to be abundant in the plasma pools due to widespread vaccination). For coating, the antigens were diluted in carbonate buffer (0.1 M, pH 9.4) to final concentrations of 1 µg/mL for the blood group A and B antigen conjugates and mannan, and 0.01% (v/v) for TT. Antigen solutions (100 µL per well) were applied to microtiter plates (FluoroNunc, Thermo Scientific Nunc A/S, Denmark) and incubated overnight at 4 °C for coating. After incubation, wells were emptied and blocked with 200 µL of TBS/Tw containing 1 g/L HSA (TBS/Tw/HSA) for 1 h at ambient temperature. Wells were then washed three times with 250 µL of TBS/Tw using an automated plate washer. Samples diluted in TBS/Tw/HSA containing 10 mM EDTA were added to the wells (100 µL per well, in duplicates) and incubated overnight at 4 °C. Subsequent steps were performed at ambient temperature. After washing, wells were incubated for 1 h with biotinylated secondary antibodies (rabbit polyclonal anti-human IgA [Dako, Denmark], IgG [Dako], or IgM [Rockland, PA, USA]) diluted to 1 mg/L in TBS/Tw/HSA.

After washing, 100 µL of europium-streptavidin complex (product 1244 − 360, PerkinElmer, MA, USA, 0.1% [v/v] in TBS/Tw containing 25 µM EDTA) was added to each well and incubated for 1 h. Wells were washed again before adding 200 µL of enhancement solution (PerkinElmer). Plates were shaken, and time-resolved fluorescence was measured after 5 min using a plate reader (VICTOR X5, PerkinElmer).

Data analysis was conducted as previously described [24]. Briefly, signals attributable to the carbohydrate moieties of glycoconjugates were estimated by subtracting signals obtained on an HSA-coated surface from those on a glycoconjugate-coated surface using the same sample. Antibody reactivity was quantified after log10 transformation of all data, with standard curves approximated by third-degree polynomials in Microsoft Excel 2016 (Microsoft Corporation, WA, USA). Data was retransformed to a linear scale for presentation.

### SDS-PAGE and western blotting

#### SDS-PAGE analysis

The concentration of affinity-purified isohemagglutinins, along with normal human (nh) IgA (I4036, Sigma-Aldrich, St. Louis, MO, USA), nhIgG (Privigen, CSL Behring, King of Prussia, PA, USA), and nhIgM (I8260, Sigma-Aldrich, St. Louis, MO, USA), was determined by spectrophotometry (NanoDrop 1000, Thermo Fisher Scientific, Waltham, MA, USA) and adjusted with TBS/Tw. Samples were then mixed with loading buffer (final concentrations: 6.5% (v/v) glycerol, 1.96% (w/v) SDS, 40 mM Tris, 5.25 M urea, 6.5 ppm (w/v) bromophenol blue, pH 6.7) and dithiothreitol (60 mM). The mixtures were boiled for 3 min. Following denaturation, samples and a protein ladder marker (Precision Plus Protein™ All Blue Prestained Protein Standards, Bio-Rad, Hercules, CA, USA) were loaded onto 4–15% gradient gels (Criterion™ TGX™, Bio-Rad, cat#5671084) and subjected to SDS-PAGE. Electrophoresis was performed at 80 V until the dye front exited the stacking gel, followed by 150 V for approximately 1 h.

#### Coomassie staining

Gels were stained with Coomassie Brilliant Blue by heating in a microwave oven for 5 min. Subsequently, gels were destained in a solution containing 9.6% (v/v) ethanol and 7.5% (v/v) acetic acid prepared in demineralized water.

#### Western blotting

Proteins were transferred onto nitrocellulose membranes using the Trans-Blot^®^ Turbo™ Transfer System (Bio-Rad) with the 7-minute transfer protocol provided by the manufacturer. Membranes were blocked in TBS with 0.1% Tween-20 for 45 min and then incubated overnight with gentle rocking in TBS/Tw/HSA containing 1 mM EDTA and biotinylated secondary antibody. The secondary antibodies against hIgG, hIgA, and hIgM were those described previously for the TRIFMAs. Biotinylated normal rabbit immunoglobulin was included as a control for nonspecific binding of biotinylated antibodies. The detection antibodies were used at a final concentration of 1 mg/L. After six washes in TBS/Tw (without azide), membranes were incubated with streptavidin-HRP conjugate (Dako, Denmark) diluted 1:3,000 in TBS/Tw (without azide) containing 1 mM EDTA and nhIgG at 0.1 g/L for 1.5 h. Signal detection was performed using SuperSignal™ West Pico chemiluminescence substrate (Thermo Fisher Scientific, Waltham, MA, USA), and images were acquired on an ImageQuant LAS 400 mini (GE Healthcare, Chicago, IL, USA).

### Bacterial strains and preparation

*S. pneumoniae* strains were obtained from the Kilian Collection, the bacterial culture collection at the Department of Biomedicine, Aarhus University, Denmark. A total of 30 serotypes were included: 1, 3, 4, 5, 6 A, 6B, 6 C, 7 F, 8, 9 N, 9 V, 10 A, 11 A, 12 F, 14, 15 A, 15B, 16 F, 18 C, 19 F, 19 A, 20, 22 F, 23 F, 23 A, 24 F, 31, 33 F, 35 F, and 38. Additionally, the unencapsulated strain CSR SCS-2 clone I, referred to as the “C-mutant” [25, 26], was included.

Individual bacterial strains were cultured overnight at 35 °C in 50 mL Todd-Hewitt broth in a 5% CO₂ heating cabinet. Cultures were harvested by centrifugation (2,000 × g, 30 min), and bacterial pellets were resuspended in PBS containing 1% (v/v) formaldehyde for fixation. After overnight incubation, the fixed bacteria were washed twice by centrifugation in 50 mL PBS, followed by two additional washes in 50 mL TBS to neutralize residual aldehyde groups. The final bacterial pellets were resuspended in TBS and stored at 4 °C until use.

### Flow cytometry

Isohemagglutinin reactivity was assessed by flow cytometry (standard configuration NovoCyte 3000, ACEA Biosciences, CA, USA). Fixed bacterial suspensions were adjusted in TBS/HSA to achieve approximately 15,000 bacterial events per µL, as determined by flow cytometry. A forward-scatter threshold was set to ensure that background events in TBS/HSA alone remained below 50 events per µL. Each recorded flow cytometry event corresponded to an average of 2.7 bacteria, based on linear regression analysis of the relationship between optical density (OD) measurements of pneumococcal suspensions (where OD 1.0 corresponds to 1.8 × 10⁹ bacteria/mL [27]) and the measured event count per µL (*R*² = 0.73). 

Fixed bacterial suspensions diluted in TBS/HSA were incubated with an equal volume of PBS/HSA containing one of the following reagents:

- No added antibodies (buffer control).
- nhIgG, 200 mg/L (positive control).
- Recombinant humanized monoclonal IgG antibody against complement C5 (eculizumab, 20 mg/L; negative control).
- Anti-A isohemagglutinin (αA-IH, 10 mg/L).
- Anti-B isohemagglutinin (αB-IH, 20 mg/L).
- Anti-A,B isohemagglutinin (αA,B-IH, 10 mg/L).
- IgG anti-αGal antibody (affinity-purified as previously described [28], 5.0 mg/L; included as a reference for previous findings)

Samples were incubated at 37 °C for 60 min, followed by two washes in 1 mL PBS/HSA (2000 × g, 10 min). The bacterial pellets were resuspended in 20 µL PBS/HSA containing 0.33% (v/v) fluorescein-isothiocyanate (FITC)-coupled polyclonal rabbit F(ab’)₂ anti-human IgG (F0315, DAKO, Denmark) and incubated in the dark at room temperature for 30 min. After incubation, 80 µL PBS was added, mixed, and 20 µL of each sample was analyzed by flow cytometry at a flow rate of 35 µL/min. Forward-scatter, side-scatter, and FITC fluorescence (excitation at 488 nm, emission detected through a 530/30 nm bandpass filter) were recorded.

To account for potential differences in bacterial background fluorescence, the median fluorescence intensity (MFI) of F(ab’)₂ anti-human IgG binding for each strain and antibody condition was normalized to the MFI of the same strain incubated without primary antibody. This ratio, MFI_norm_, has a theoretical value of 1.0 in the absence of primary antibody binding.

### Inhibition experiments

To evaluate the inhibition of isohemagglutinin binding to solid-phase ABO antigens by RBCs, isohemagglutinin preparations were diluted in sample buffer (αA-IH, 1:10,000; αB-IH, 1:7,500) containing glutaraldehyde-fixed type A or B RBCs at varying hematocrit levels. The mixtures were incubated for 30 min at ambient temperature, followed by 4 h at 4 °C with end-over-end rotation. After incubation, samples were centrifuged at 200 × g for 5 min, and the supernatants were analyzed for residual reactivity with blood group A or B antigens using TRIFMA.

For inhibition experiments analyzed by flow cytometry, the isohemagglutinin concentrations were adjusted for each bacterial strain to achieve an MFI_norm_ of approximately 5 in the absence of an inhibitor. As soluble inhibitors, we used pneumococcal capsule polysaccharides of vaccine quality from serotypes 6B, 7 F, and 9 V (LCG Standards, Teddington, UK), as well as pneumococcal Cell Wall Polysaccharide, CWP (Statens Serum Institut, Copenhagen, Denmark). Soluble polysaccharide inhibitors were incubated with isohemagglutinins for one hour at 37 °C before being added to fixed bacterial cells. The subsequent handling and flow cytometry analysis proceeded as described above.

The percentage of inhibition was determined using standard curves generated from serial dilutions of the tested isohemagglutinin preparation, analyzed alongside the inhibition experiments for each bacterial strain. Quantitative calculations were performed as described for the TRIFMA assay.

### Cohort establishment and data sources

To investigate any association between ABO blood group and invasive pneumococcal disease (IPD), we established a nationwide cohort comprising the entire Danish population (approximately 6 million inhabitants as of 2025). ABO blood group data together with RhD antigen status (included as a control) were obtained from all Danish blood bank computer systems, where each record was linked to the unique personal identification number (CPR) assigned to all Danish residents since 1968. Duplicate entries were removed, and only individuals with a unique CPR number were included in the final cohort.

Data on IPD were obtained from the Statens Serum Institut (Copenhagen, Denmark), which performs nationwide surveillance of IPD and serotyping of clinical isolates. The dataset spanned the period from 1966 to June 26, 2014; however, only IPD cases with a documented CPR number were included in the analysis. Furthermore, IPD events occurring in individuals under 2 years of age were excluded, as anti-A and anti-B antibodies are typically not expected to be fully formed in young children. The IPD and ABO blood group datasets were subsequently merged via the CPR identifier for downstream analyses.

### Statistics

Effect sizes were estimated with 95% confidence intervals (CIs) using bootstrapping with 5,000 iterations, as implemented in Estimation Statistics [29].

Data on ABO blood group types and IPD events were imported and joined in an Oracle SQL database, using the CPR identifier as the primary key. The number of IPD cases for specific pneumococcal serotypes was stratified by ABO blood group type using STATA version 12 (StataCorp, College Station, TX, USA).

GraphPad prism version 10 for Windows (GraphPad Software, Boston, MA, USA) was used for regression analyses, curve fitting (including Hill-slope calculations), odds ratio estimations, and figure preparations.

The standard deviation (${\sigma }_{z}$) of a product ($z=x\cdot y$) was calculated using the error propagation formula for multiplication:

$$ {\sigma }_{z}=z\cdot \sqrt{{\left(\frac{{\sigma }_{x}}{x}\right)}^{2}+{\left(\frac{{\sigma }_{y}}{y}\right)}^{2}}$$

where $$ {\sigma }_{x}$$​ and $$ {\sigma }_{y}$$​ are the standard deviations of $$ x$$ and $$ y$$, respectively.

### Ethics

Anonymized blood samples used in this study consisted of surplus material from voluntary blood donations collected at the blood bank of Aarhus University Hospital. The use of these samples complied with current Danish legislation. Approval for the registry study was obtained from the Danish Data Protection Agency (Ref. no. 2014-41-3065).

## Results

### Isohemagglutinin affinity purification

Isohemagglutinins were purified by affinity chromatography from pooled human plasma using beads conjugated with ABO antigens, followed by acidic elution. Plasma from ABO type B donors (containing anti-A isohemagglutinins) was passed through a blood group A antigen column, yielding ‘αA-[B]’ eluates. Similarly, plasma from ABO type A donors (containing anti-B isohemagglutinins) was processed using a blood group B antigen column to generate ‘αB-[A]’ eluates. Plasma from ABO type O donors (containing both anti-A and anti-B isohemagglutinins) was processed in two separate batches: one passed through a blood group A antigen column to yield ‘αA-[O]’ eluates and the other through a blood group B antigen column to yield ‘αB-[O]’ eluates.

**Fig. 1:**
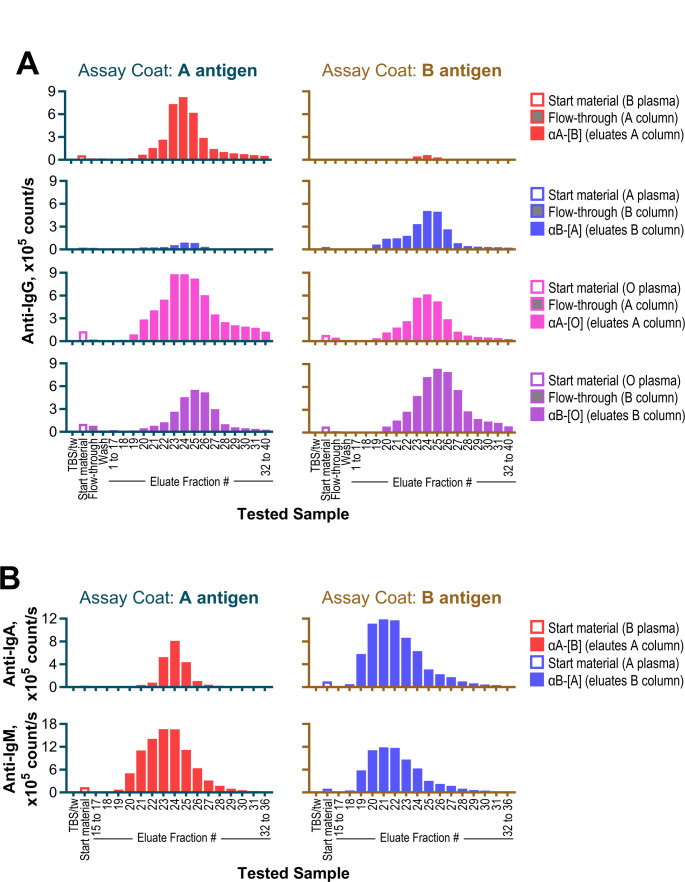
Affinity purification and detection of anti-A and -B isohemagglutinins. **A** Histograms depicting signals from time-resolved immunofluorescence assays (TRIFMA) for IgG isohemagglutinins in eluates obtained through affinity purification. Testing was performed on A-antigen- and B-antigen-coated surfaces (left and right panels, respectively). For comparison, signals were also measured in the dilution buffer (TBS/tw), starting materials (plasma pools from ABO type B, A, and O donors), flow-through fractions (10 × 50 mL, averaged for simplicity), and wash buffer. For each procedure, the coefficient of variation for the 10 flow-through fraction signals did not exceed 9%. **B** Histograms showing TRIFMA signals for IgA (top panels) and IgM (bottom panels) isohemagglutinins in selected αA-[B] eluates tested on A-antigen-coated surfaces (left panels) and αB-[A] eluates tested on B-antigen-coated surfaces (right panels), alongside their corresponding starting materials and the dilution buffer

Affinity-purified eluates, along with their corresponding plasma pools, flow-through fractions, and wash buffers, were analyzed for IgG antibodies against blood group A and B antigens using TRIFMAs. On a blood group A antigen-coated surface, αA-[B] eluates (fractions 20–40) showed signals comparable to or exceeding those of the starting material, with peak signals in fractions 23–25 (Fig. 1A). Minimal or no signal was detected on the blood group B antigen-coated surface, confirming the expected specificity. Flow-through and wash fractions also showed minimal signals. Similar results were observed for αB-[A] eluates tested on blood group B antigen-coated surfaces, with peak signals in fractions 23–26.

For αA-[O] and αB-[O] eluates, the predominant reactivity was observed toward the corresponding antigens used for purification; however, substantial reactivity with the other blood group antigen, not used for purification, indicated co-purification of anti-A and anti-B isohemagglutinins. This suggests that αA-[O] and αB-[O] eluates contained mixtures of anti-A, anti-B, and anti-A, B isohemagglutinins; apparently, the latter antibodies are reactive to both blood group A and B antigens.

To assess potential co-elution of IgA and IgM isohemagglutinins, selected αA-[B] and αB-[A] eluates were analyzed for these immunoglobulin classes. IgA and IgM signals were detected in the same fractions as IgG (Fig. 1B), indicating co-elution.

For subsequent analyses, eluate fractions were pooled into three isohemagglutinin preparations:

- **αA-IH**: αA-[B] fractions 20–28 (protein concentration: 0.31 g/L).
- **αB-IH**: αB-[A] fractions 19–27 (protein concentration: 0.36 g/L).
- **αA**,**B-IH**: a combined pool of αA-[O] fractions 19–29 and αB-[O] fractions 20–30 (protein concentration: 0.28 g/L).

### Isohemagglutinin characterization

**Fig. 2:**
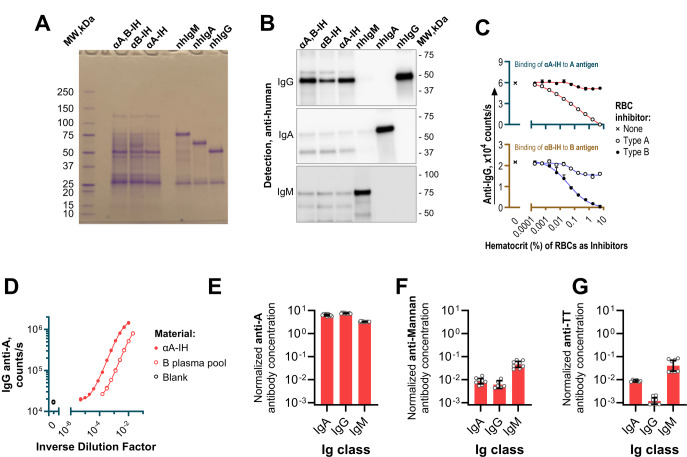
Characterization of Isohemagglutinin preparations. **A** SDS-PAGE analysis of the three isohemagglutinin preparations alongside purified nhIgG, nhIgA, and nhIgM for comparison. The wells were loaded with αA, B-IH (6 µg), αB-IH (8 µg), αA-IH (4 µg), nhIgG (1 µg), nhIgA (1 µg), and nhIgM (2 µg). The gel was stained with Coomassie Brilliant Blue to visualize the protein bands. A band around ~ 25 kDa, present in all samples, corresponds to immunoglobulin light chains **B** Cropped western blot analysis of isohemagglutinin preparations. The wells were loaded with αA, B-IH (0.5 µg), αB-IH (1 µg), αA-IH (0.5 µg), nhIgG (0.2 µg), nhIgA (0.2 µg), and nhIgM (0.2 µg). Blots were developed using rabbit anti-human IgG (top), IgA (middle), and IgM (bottom). Uncropped blots and control experiments using normal rabbit immunoglobulin for detection are provided in Fig. S1. **C** The ability of RBCs to inhibit antibody binding to ABO-antigen-coated surfaces was assessed. Upper panel: The αA-IH preparation (1:10,000 dilution in sample buffer) was incubated with type A or B RBCs at varying hematocrit levels and tested for IgG binding to an A-antigen-coated surface. Lower panel: The αB-IH preparation (1:7,500 dilution) was similarly tested on a B-antigen-coated surface. Data are presented as mean IgG binding signals, with the sample buffer-only signal subtracted, and standard deviations from duplicate experiments as a function of RBC hematocrit. **D** IgG binding to blood group A antigen as a function of relative antibody concentration. Dilution series of two materials were tested: αA-IH (affinity-isolated anti-A antibodies) and its starting material (ABO type B plasma pool diluted in TBS/Tw). The x-axis shows the inverse dilution factor on a log₁₀ scale, representing relative antibody concentration. The parallel binding curves suggest similar avidity of anti-A antibodies in both preparations. **E–G** Histograms showing anti-A **E**, anti-mannan **F**, and anti-TT **G** antibody levels in αA-IH, expressed relative to their corresponding levels in the starting material (ABO type B plasma pool) across different immunoglobulin classes. Samples were analyzed by TRIFMA, with bars representing the mean of repeated measurements and error bars indicating 95% confidence intervals

The purity of the three isohemagglutinin preparations was assessed by reduced SDS-PAGE. Purified nhIgG, nhIgA, and nhIgM were included as reference proteins. We found that all three preparations displayed a dominant band at ~ 50 kDa, corresponding to the heavy chain of nhIgG (Fig. 2A), with only faint bands at ~ 65–70 kDa (heavy chain nhIgA) and ~ 75 kDa (heavy chain nhIgM). A band at ~ 25 kDa, present in all samples, corresponds to immunoglobulin light chains. Western blot analysis using isotype-specific antibodies for human IgG, IgA, and IgM confirmed these findings (Fig. 2B and Fig. S1), further supporting that IgG is the predominant immunoglobulin class in the hemagglutinin preparations. Absolute quantification of immunoglobulin class distribution was not performed.

To further assess antigen specificity, αA-IH and αB-IH binding to solid-phase blood group A and B antigens, respectively, was assessed in the presence of increasing concentrations of type A and B RBCs. We confirmed that αA-IH IgG binding to blood group A antigen was efficiently inhibited by increasing concentrations of type A RBCs, with only minor inhibition by type B RBCs, and vice versa for αB-IH (Fig. 2C). Comparable results were obtained for IgA and IgM isohemagglutinins (Fig. S2), further validating the specificity of the affinity purification procedure.

To further evaluate the binding properties of the isolated hemagglutinins, we performed dilution series of αA-IH and the ABO type B plasma pool used for purification, measuring the IgG anti-A binding signal by TRIFMA. We found that the resulting binding curves were parallel (Fig. 2D), indicating that the avidity of IgG anti-A in αA-IH was comparable to that in the original plasma pool. Similar findings were obtained for IgA and IgM anti-A (Fig. S3) and for anti-B antibodies in αB-IH compared to the ABO type A plasma pool (Fig. S4), confirming that the antibody binding properties to ABO antigens remained intact.

Affinity purification efficiency was evaluated by quantifying antibodies in αA-IH, expressed relative to their corresponding levels in the starting material (ABO type B plasma pool). TRIFMAs were used to measure IgA, IgG, and IgM antibodies against blood group A antigen, as well as mannan and TT, which served as controls for nonspecific binding. Anti-A antibodies were highly enriched in αA-IH, whereas anti-mannan and anti-TT antibodies were detectable at significantly lower levels: approximately 1,000- to 10,000-fold lower for IgG (Fig. 2E–G). Similar results were found for αB-IH (Fig. S5).

The low level of contaminant antibody showed a consistent pattern across immunoglobulin classes: highest in IgM, intermediate in IgA, and lowest in IgG. This might reflect the antigen-binding valency of each class. For example, small antibody subsets within the isolated isohemagglutinins may exhibit additional reactivity with mannan or TT. IgM, with ten antigen-binding sites, is most likely to achieve sufficient avidity for such interactions. In contrast, IgA, present as both monomers (two binding sites) and dimers (four binding sites), shows intermediate levels, while IgG, with only two binding sites, has the lowest potential for such additional reactivity. However, this possibility was not explored further in this study.

Analysis of antibody recovery revealed that approximately half the loaded anti-A and anti-B antibodies were retained in αA-IH and αB-IH, respectively, with only trace amounts remaining in flow-throughs (Fig. S6). Additionally, anti-mannan and anti-TT antibodies were nearly absent in the eluates and remained in the flow-through fractions.

These results confirmed that the affinity purification procedure effectively isolated the specific isohemagglutinins while minimizing the presence of other antibody specificities.

### Isohemagglutinin polyreactivity against *S. pneumoniae*

#### Selection of S.pneumoniae serotypes

To investigate isohemagglutinin polyreactivity, we selected encapsulated *S. pneumoniae* as model organisms based on their structural diversity, clinical significance, and suitability for studying antibody polyreactivity. To ensure clinical relevance, we analyzed Danish national surveillance data on pneumococcal serotypes identified from sterile compartments in patients older than 2 years (allowingfor full isohemagglutinin development). Data for patients suffering from IPD inthe period 1966 to 2014 and with known ABO blood group type was analyzed. The30 most frequently detected serotypes, collectively accounting for 96% of allcases, were selected for the study (Table 1).

**Table 1:** Frequency of invasive Pneumococcal disease by serotype of the isolated organism in Danish patients aged > 2 years with known ABO blood group (1966–2014)

Serotype	Cases	Proportion (%)	Cumulative proportion (%)
1	1946	13.8	13.8
7 F	1041	7.40	21.2
4	995	7.07	28.3
14	946	6.72	35.0
3	816	5.80	40.8
8	772	5.49	46.3
9 V	679	4.83	51.1
12 F	671	4.77	55.9
22 F	500	3.55	59.5
23 F	490	3.48	62.9
9 N	472	3.35	66.3
19 A	420	2.98	69.3
19 F	420	2.98	72.3
6B	379	2.69	75.0
6 A	333	2.37	77.3
18 C	266	1.89	79.2
11 A	265	1.88	81.1
33 F	213	1.51	82.6
20	208	1.48	84.1
24 F	188	1.34	85.4
38	161	1.14	86.6
10 A	151	1.07	87.6
23 A	139	0.988	88.6
35 F	134	0.952	89.6
16 F	126	0.895	90.5
5	124	0.881	91.4
15 A	120	0.853	92.2
6 C	108	0.768	93.0
15B	106	0.753	93.7
31	87	0.618	94.4
’Rough’	134	0.952	95.3
Others	661	4.70	100
**Total**	14,071	100	—

Data from the Danish national surveillance registry for invasive pneumococcal disease. ‘Rough’ refers to unencapsulated pneumococci. ‘Others’ include various less common serotypes and strains with uncertain serotype designation. Data for all hosts, including those younger than two years of age, are presented in Table S1

#### Reactivity of isohemagglutinins with *S. pneumoniae*

**Fig. 3:**
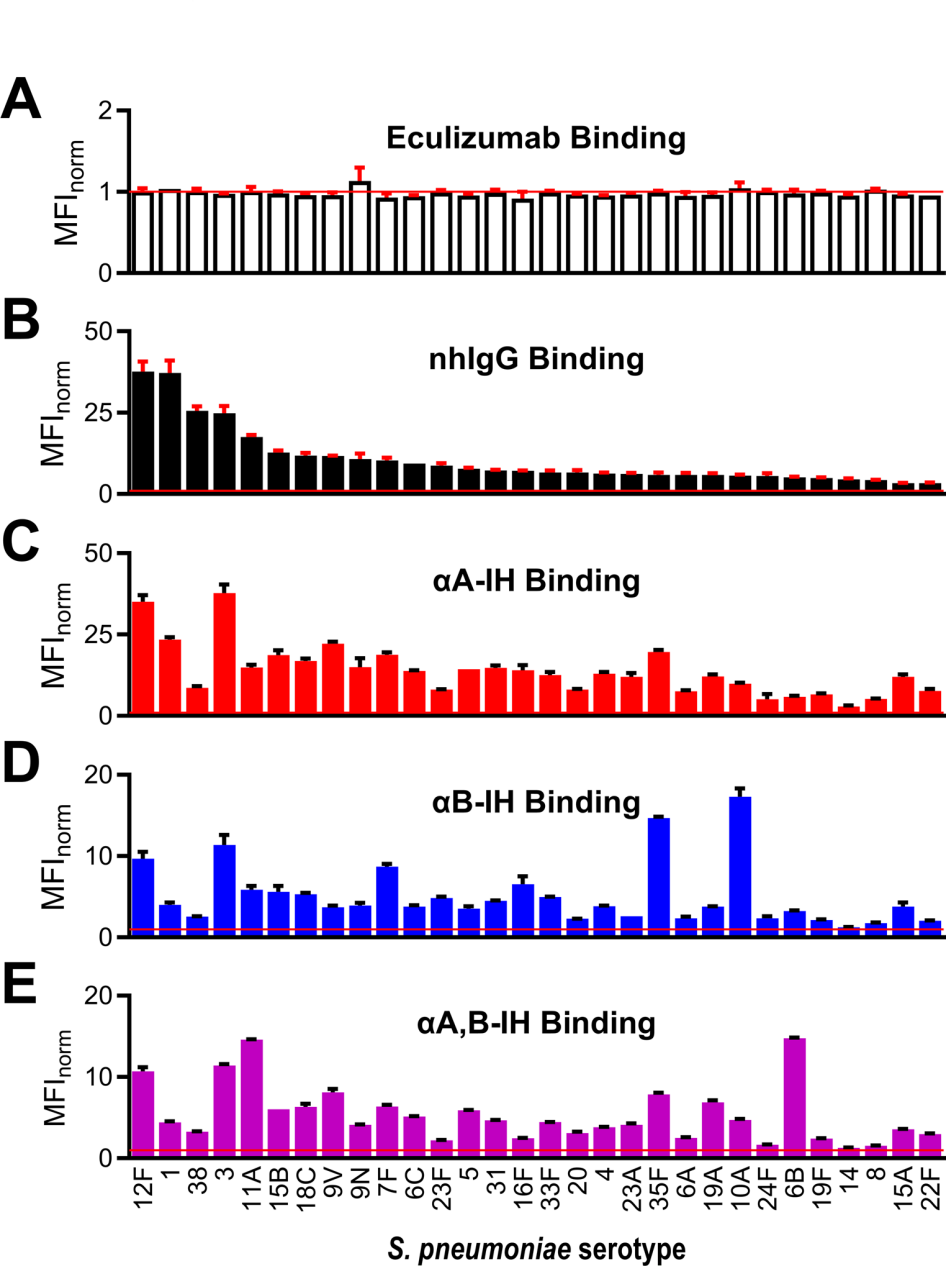
Binding of antibody preparations to encapsulated *S. pneumoniae* strains. The binding of antibodies from isohemagglutinin preparations to 30 pneumococcal strains of different serotypes was assessed using flow cytometry with fluorescently labeled F(ab’)₂ anti-human IgG for detection. **A** Control antibody preparations included eculizumab (negative control) at 20 mg/L and **B** nhIgG (positive control) at 200 mg/L. Isohemagglutinin preparations included αA-IH at 10 mg/L (**C**), αB-IH at 20 mg/L (**D**), and αA, B-IH at 10 mg/L (**E**). The bars represent the mean MFI_norm_ of two independent experiments, with error bars indicating standard deviations. Note that y-axis scales vary across panels

 The binding of IgG isohemagglutinins to formaldehyde-fixed pneumococci was detected using flow cytometry with fluorescently labeled F(ab’)₂ anti-human IgG. To define the threshold for positive binding, eculizumab (an irrelevant recombinant humanized monoclonal IgG antibody against human complement protein C5) was included as a negative control. Binding was quantified by calculating the fluorescence signal from eculizumab at 20 mg/L relative to the background signal obtained without primary antibody for each strain. This unitless ratio, MFI_norm_, had a mean of 0.98 (95% CI: [0.97,1.0]) across strains representing the 30 different serotypes, ranging from 0.92 to 1.1 (Fig. 3A). Based on these results, we defined positive IgG binding as a mean MFI_norm_ at least two standard deviations above 1.1.

As a positive control, nhIgG, expected to contain pneumococcal-reactive antibodies, was tested at 200 mg/L, and it bound to all 30 pneumococcal strains (Fig. 3B). The isohemagglutinin preparations were tested at concentrations corresponding to ~ 25% of the average IgG isohemagglutinin activity present in the starting material used for their affinity purification: 10 mg/L for αA-IH and αA, B-IH, and 20 mg/L for αB-IH. These concentrations reflect the degree of enrichment achieved during affinity purification. Specifically, IgG anti-A in αA-IH was enriched ~ 8-fold compared to its starting material (the B plasma pool) (Fig. 2E). Accordingly, αA-IH was used at 3.1% of the incubation volume (3.1% × 8 ≈ 25%). Likewise, IgG anti-B in αB-IH was enriched ~ 5-fold relative to its starting material (the A plasma pool) (Fig. S5) and was therefore used at 5.0% of the incubation volume (5% × 5 ≈ 25%). Notably, all three isohemagglutinin preparations demonstrated binding to all 30 serotypes tested (Fig. 3C–E), but binding signals tended to be higher for αA-IH compared with αB-IH and αA, B-IH (note the different scaling in Fig. 3C versus Fig. 3D–E). Thus, when normalized to approximate physiological activity levels, αA-IH exhibited higher binding signals than αB-IH and αA, B-IH, reflecting both differences in enrichment efficiency and baseline reactivity.

Contaminating antibodies were unlikely to explain this reactivity, as their levels were reduced by at least 1000-fold relative to isohemagglutinins in the preparations. Isohemagglutinin preparations were tested at 10 or 20 mg/L, whereas nhIgG, even at a 100-fold lower concentration (0.1–0.2 mg/L), showed no detectable binding, as seen in Fig. S7.

### Isohemagglutinins target pneumococcal Cell Wall Polysaccharide (CWP)

The consistent binding of isohemagglutinins to pneumococcal strains of diverse serotypes suggests the presence of a conserved antigen accessible across all tested strains. A likely candidate is pneumococcal cell wall polysaccharide (CWP), a ubiquitous structural component of *S. pneumoniae* [26].

**Fig. 4:**
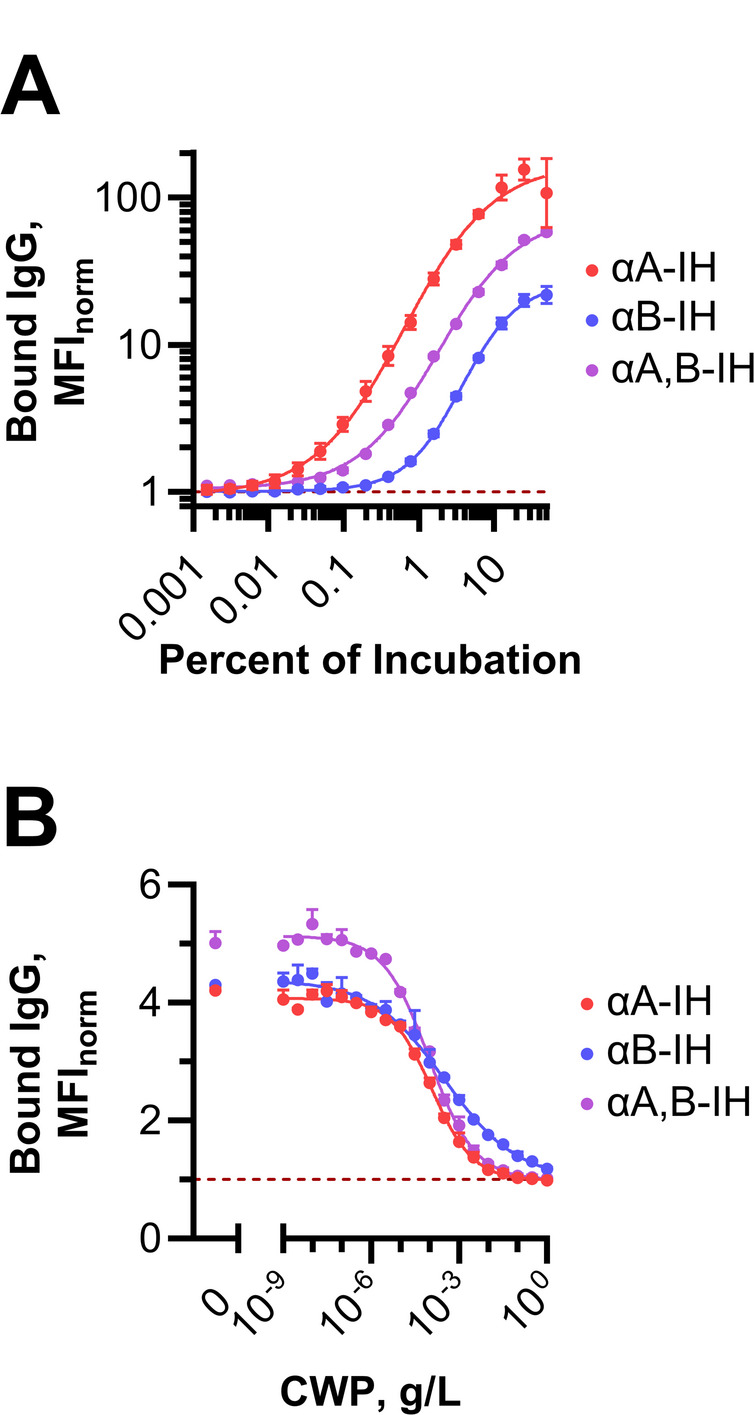
Binding of isohemagglutinin preparations to the CWP-covered C-mutant. **A** Each of the three isohemagglutinin preparations was tested at varying concentrations for binding to the pneumococcal strain C-mutant, which is entirely covered by a dense capsular-like structure composed of CWP. Binding was detected using fluorescently labeled F(ab’)₂ anti-human IgG and analyzed by flow cytometry, with normalized median fluorescence intensity (MFI_norm_) as the readout. Data represents the mean and standard deviation (SD) from three independent experiments, fitted with a sigmoidal curve. The Hill slopes were αB-IH (1.1, 95% CI: 1.1–1.2) >αA, B-IH (0.81, 95% CI: 0.77–0.86) >αA-IH (0.73, 95% CI: 0.63–0.83), indicating that αB-IH exhibited the strongest avidity for the C-mutant. The potential influence of other undetected immunoglobulin classes on these results cannot be excluded. **B** Similar experiments were conducted using fixed concentrations of each isohemagglutinin preparation while varying soluble CWP as a competitive inhibitor. Isohemagglutinin concentrations were adjusted in preliminary experiments to yield an MFI_norm_ of four to five in the absence of CWP inhibition. High CWP concentrations (1 g/L) were required to abolish binding. The Hill slopes for inhibition were in the reverse order: αB-IH (– 0.35, 95% CI: – 0.43 to – 0.27) < αA, B-IH (– 0.56, 95% CI: – 0.61 to – 0.50) < αA-IH (– 0.62, 95% CI: – 0.68 to – 0.55), further supporting the strongest avidity of αB-IH for C-mutant. Again, the influence of other undetected immunoglobulin classes cannot be excluded

To evaluate this hypothesis, we assessed isohemagglutinin binding to the C-mutant: a pneumococcal strain that lacks an actual capsule but exhibits a particularly thick CWP-rich layer [30]. Notably, IgG antibodies from all three isohemagglutinin preparations bound to the C-mutant (Fig. 4A), despite the fact that the CWP does not contain ABO antigens [31]. Furthermore, this binding was inhibited in a dose-dependent manner by increasing concentrations of soluble CWP prepared from the same strain (Fig. 4B), indicating that isohemagglutinins bind pneumococcal CWP despite the absence of ABO antigens.

### Anti-A isohemagglutinins target pneumococcal capsular polysaccharide serotypes 7F and 9V

To determine whether isohemagglutinins exhibit specific reactivity toward pneumococcal capsular polysaccharides (CPS), we assessed the inhibition of anti-A isohemagglutinin binding to pneumococci of serotypes 6B, 7F, and 9V using their respective CPSs as soluble inhibitors in a checkerboard assay. These strains exhibited low to intermediate reactivity with αA-IH (Fig. 3). The C-mutant was included as a control strain, and CWP served as a control inhibitor.

**Fig. 5:**
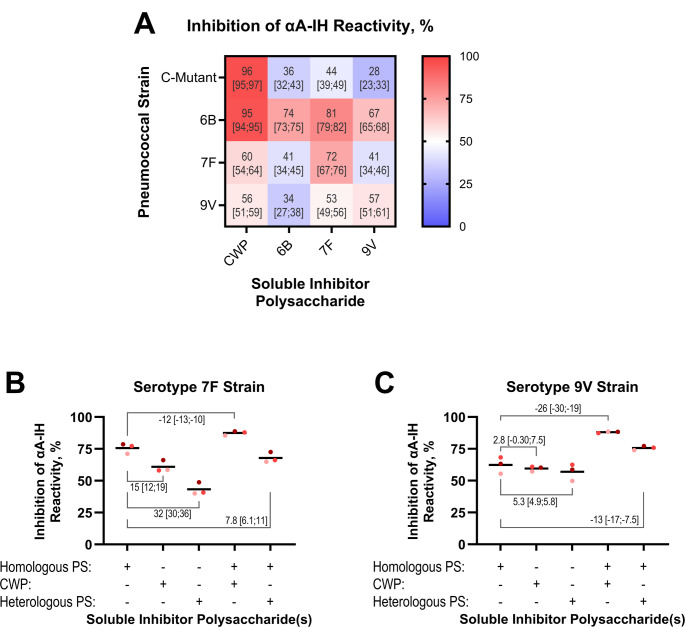
Inhibition of αA-IH reactivity with pneumococcal strains by soluble polysaccharides. **A** Heatmap and values representing the percent inhibition of αA-IH binding to four different pneumococcal strains by four different polysaccharides. Each polysaccharide was preincubated at a final concentration of 100 mg/L with the αA-IH preparation, which was used at a concentration yielding an MFI_norm_ of approximately 5 for the specific strain in the absence of inhibition. The mixture was then incubated with the fixed bacterial strain and bound IgG was then measured using fluorescently labeled F(ab’)₂ anti-human IgG and flow cytometry. For each strain, a serial dilution of the αA-IH preparation was analyzed in parallel to quantify the inhibited reactivity. Values represent the mean inhibition with 95% confidence intervals (CI) in brackets, based on three independent experiments conducted on separate days. **B** Inhibition of αA-IH binding to a serotype 7 F strain, following the same approach as in panel (**A**), but also including experiments with combined homologous CPS and CWP inhibition as well as combined heterologous CPS and CWP inhibition. Data points from experiments conducted on the same day are shaded in similar red tones. The average difference in inhibition relative to that achieved with homologous CPS alone was calculated using a paired bootstrapping approach to estimate the 95% CI. **C** As in panel (**B**), but for a serotype 9 V strain

As expected, CWP abolished αA-IH binding to C-mutant (Fig. 5A and Fig. S8). However, CPS6B, CPS7F, and CPS9V also partially inhibited αA-IH binding to C-mutant, likely due to contaminating CWP [32], which is covalently linked to the CPS from most pneumococcal serotypes [33].

CPS6B partially inhibited binding to its homologous bacterial strain, but the inhibition levels were comparable to those observed with heterologous CPS7F and CPS9V, suggesting that αA-IH binding to this strain was primarily mediated by CWP interactions. In agreement, CWP abolished αA-IH binding to pneumococci of serotype 6B. However, CPS6B inhibited binding to the homologous serotype 6B strain approximately twice as effectively as to the C-mutant or pneumococci of serotypes 7F and 9V, suggesting some reactivity unique to CPS6B. This might be explained by a subset of anti-A isohemagglutinins with dual reactivity toward both CPS6B and CWP, in addition to the blood group A antigen.

In contrast, CPS7F was the most effective inhibitor for pneumococci of serotype 7F, followed by CWP, whereas CPS6B and CPS9V preparations showed weaker inhibition, suggesting selective αA-IH targeting of CPS7F. For the serotype 9V strain, inhibition by CPS9V was only slightly stronger than that of CWP, suggesting mixed interactions with CPS and CWP.

To further delineate the contribution of reactivity with CPS and CWP, we performed additional inhibition experiments using combinations of CWP with either homologous or heterologous CPSs for pneumococci of serotypes 7F and 9V. In both cases, inhibition was more pronounced when homologous CPS and CWP were combined in comparison with heterologous CPS-CWP combinations or homologous CPS alone (Fig. 5B). These findings support the existence of distinct isohemagglutinin subsets: some target specific pneumococcal CPSs, others bind CWP, and some might cross-react with multiple polysaccharides such as both CPS6B and CWP.

### Isohemagglutinins react with select pneumococcal serotypes

To comprehensively assess isohemagglutinin reactivity toward pneumococcal CPS, we adsorbed out CWP cross-reactive antibodies from our preparations and subsequently evaluated the residual binding to pneumococci representing the 30 serotypes by flow cytometry. Soluble CWP was applied at concentrations empirically determined to abolish reactivity with the C-mutant strain (Fig. 4B).

**Fig. 6:**
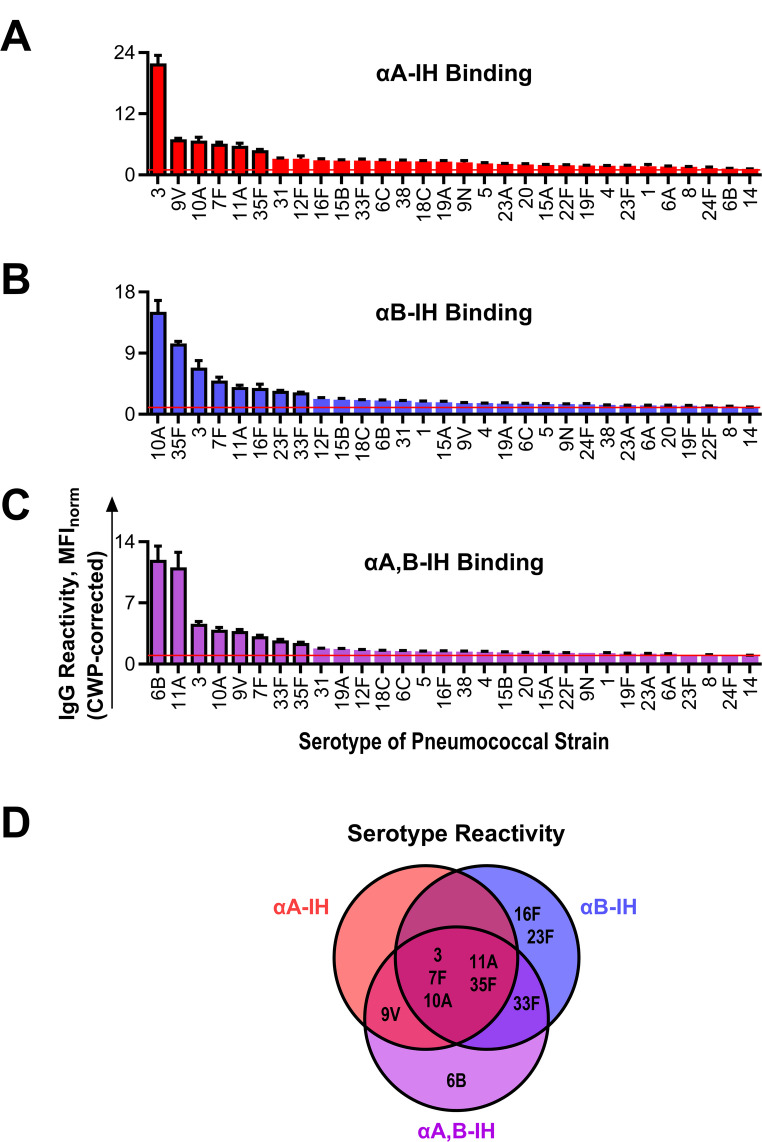
Isohemagglutinin reactivity with pneumococcal serotypes. **A–C** Corrected binding signals (MFI_norm_) for the three isohemagglutinin preparations—αA-IH **(A)**, αB-IH **(B)**, and αA, B-IH **(C)**—against pneumococci representing 30 different serotypes. Data are derived from those shown in Fig. 3 and have been adjusted to account for non-serotype-specific binding mediated by the CWP. The contribution of CWP to the total signal for each strain was estimated from the proportion of binding lost following pre-adsorption with soluble CWP (Fig. S9 and S10). Bars represent the mean of the product between the uncorrected binding signal and the fraction of signal remaining after CWP adsorption. Error bars denote standard deviation, calculated using the propagation of uncertainty for multiplication. Serotypes with substantial residual binding—defined pragmatically—are highlighted with black-bordered bars. **D** Venn diagram illustrating the overlap and specificity of serotype binding across the three isohemagglutinin preparations, based on the subsets of serotypes with substantial residual binding shown in panels A–C

Following adsorption with CWP, isohemagglutinin binding signals to the pneumococci were reduced by 14–98% (Fig. S9 and S10). To correct for CWP-mediated reactivity, the crude binding signals presented in Fig. 3 were adjusted based on the proportion of reactivity attributable to CWP binding for each strain (Fig. S10). This correction markedly diminished apparent reactivity for most strains. However, a subset of approximately 20–25% of the serotypes retained substantial binding with each preparation, whereas the remainder exhibited minimal or no residual binding (Fig. 6A–C). Notably, none of the serotypes with substantial residual binding is known to express ABO antigens [9, 10], supporting polyreactivity as the basis for these interactions.

Notably, the three isohemagglutinin preparations showed partial overlap in reactivity for serotypes, demonstrating substantial binding but also subtle differences. For example, serotypes 3, 7F, 10A, 11A, and 35F were bound by all three preparations, whereas serotype 9V was exclusively bound by αA-IH and αA, B-IH, and serotype 6B was bound only by αA, B-IH (Fig. 6D).

These findings highlight the heterogeneity of naturally occurring isohemagglutinins and their differential recognition of select pneumococcal CPS antigens, extending well beyond their CWP reactivity.

### Limited protective effect of isohemagglutinins against invasive pneumococcal disease

To investigate whether isohemagglutinins may confer protection against IPD, we conducted a nationwide analysis using data from the entire Danish population. ABO blood group types were used as proxies for the presence of specific isohemagglutinins, based on the inverse relationship between ABO antigen expression and the corresponding naturally occurring antibodies.

We identified 2,597,659 individuals with known ABO blood types and linked this information, using the Danish personal identification number, to national records of laboratory-confirmed, serotype-specific IPD diagnoses. Of these, 14,071 individuals had a registered IPD event after the age of two years; a lower age limit chosen to minimize the inclusion of individuals with immature isohemagglutinin production.

**Fig. 7:**
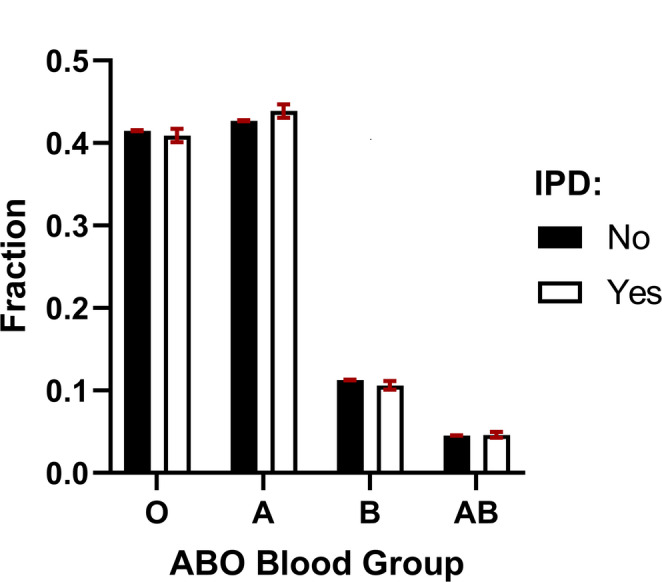
Distribution of ABO blood types in individuals with and without verified IPD. The figure includes all available data from individuals residing in Denmark between 1968 and 2024. Bars represent point estimates of ABO blood group frequencies, with 95% confidence intervals calculated using binomial distributions. Statistically, blood group A was slightly more common among IPD cases than non-cases (OR = 1.05; 95% CI: 1.00–1.10), while blood group B was slightly less common (OR = 0.93; 95% CI: 0.88–0.98). No statistically significant differences were observed for blood groups O or AB

The distribution of ABO blood types was essentially similar across individuals with and without IPD (Fig. 7). Notably, individuals with blood group AB (who do not produce isohemagglutinins) did not exhibit an increased frequency of IPD. Their odds ratio (OR) for IPD was 1.0 (95% CI: 0.95–1.1) compared with type O individuals, 0.99 (95% CI: 0.91–1.1) compared with type A, and 1.1 (95% CI: 0.99–1.2) compared with type B.

To further explore potential serotype-specific protection, we applied the isohemagglutinin–serotype binding profiles from Fig. 6A–C to stratify IPD cases by serotypes that retained substantial binding after correction for CWP-mediated reactivity. For statistical robustness, serotypes were grouped by shared isohemagglutinin reactivity patterns (Fig. 6D), and within each group, we compared the odds of IPD among individuals lacking the relevant isohemagglutinin (due to ABO type) with those capable of producing it.

Among the five defined serotype reactivity groups, only one showed a statistically significant association with ABO type after correction for multiple comparisons (Table 2). Individuals of blood group A or AB had an increased risk of IPD caused by serotype 9V compared with those of type O or B (OR 1.2; 95% CI: 1.1–1.4), consistent with the observed reactivity of anti-A and anti-A,B isohemagglutinins toward this serotype.

**Table 2:** Association between ABO blood group and risk of invasive Pneumococcal disease caused by serotypes with substantial isohemagglutinin reactivity

Serotype(s)	Reactive isohemagglutinin	Exposed (non-producers of reactive isohemagglutinin)				Unexposed (producers of reactive isohemagglutinin)				Odds ratio (95%CI)	*P* (Bonf.)
		ABO-type(s)	IPD (*n*)	No IPD (*n*)	Total (*n*)	ABO-type(s)	IPD (*n*)	No IPD (*n*)	Total (*n*)		
16 F and 23 F	Anti-B	B, O, and AB	343	1,487,775	1,488,118	A	273	1,108,663	1,108,936	0.94 (0.80–1.1)	1.0
6B	Anti-A,B	A, B, and AB	203	1,519,261	1,519,464	O	176	1,077,414	1,077,590	0.82 (0.67–1.0)	0.25
33F	Anti-B and -A,B	B and AB	36	410,492	410,528	A and O	177	2,186,349	2,186,526	1.1(0.76–1.6)	1.0
** 9V**	**Anti-A and -A**,**B**	**A and AB**	**356**	**1**,**226**,**129**	**1**,**226**,**485**	**B and O**	**322**	**1**,**370**,**247**	**1**,**370**,**569**	**1.2 (1.1–1.4)**	**0.029**
3, 7F, 10A, 11A, and 35F	Anti-A, -B, and -A,B	AB	98	117,451	117,549	A, B, and O	2306	2,477,199	2,479,505	0.90 (0.73–1.1)	1.0

‘Reactive isohemagglutinin’ refers to the isohemagglutinin preparations used in the present study: ‘anti-A’ for αA-IH reactivity, ‘anti-B’ for αB-IH reactivity, and ‘anti-A, B’ for αA, B-IH reactivity. Based on the presumed protective effect of isohemagglutinins against reactive pneumococcal serotypes, individual categorized as ‘Exposed’ had ABO types incompatible with production of the reactive isohemagglutinin, whereas ‘Unexposed’ individuals had ABO types compatible with the production of the reactive isohemagglutinin. P-values were adjusted for multiple testing using the Bonferroni correction (m = 5). Corrected p-values were calculated as *P* (Bonf.) = *P*_*raw*_×*m*, with a cap at 1.0

Exploratory, uncorrected analyses of individual serotypes stratified by ABO group suggested that, in addition to serotype 9V, individuals with blood group A may have a modestly increased risk (10%–30%) of IPD caused by serotypes 1, 7F, and 19A (Fig. S11). However, these associations did not remain significant after correction for multiple comparisons and should be interpreted with caution.

In summary, these findings suggest that isohemagglutinins exert, at most, a limited protective effect against IPD, with the clearest association being a modest inverse relationship between serotype 9V and the capability to produce anti-A or anti-A, B isohemagglutinins.

## Discussion

In this study, we demonstrate that isohemagglutinins—naturally occurring antibodies known for their specificity toward ABO blood group antigens—also exhibit synergistic polyreactivity toward surface polysaccharides of *S. pneumoniae*. These antibodies retain their reactivity toward ABO antigens while also binding to pneumococcal strains across 30 diverse serotypes. This binding is largely mediated by conserved CWP, but inhibition and adsorption experiments allow us to hypothesize that distinct clones can also bind specific CPSs lacking ABO-like structures. These findings provide strong evidence of synergistic polyreactivity and reinforce the concept that antigen-defined antibodies can harbor broader specificities.

Despite this reactivity, our epidemiological analysis revealed only a modest reduction in the occurrence of IPD caused by a single serotype (9V) among individuals with blood groups O and B—those capable of producing serotype 9V reactive isohemagglutinins. Additionally, individuals with blood group A may have a slightly increased risk of IPD caused by serotypes beyond 9V. This implies a limited functional impact of such antibody reactivity on IPD risk.

### Interpretation and implications of synergistic polyreactivity

Our findings significantly extend previous observations of synergistic polyreactivity in anti-αGal antibodies [14]. The ability of isohemagglutinins to bind both common and type-specific pneumococcal polysaccharide antigens supports a model in which naturally occurring antibodies operate collectively against diverse microbial targets. This may also explain previously reported isohemagglutinin binding to gram-negative gut bacteria [21].

### Mechanistic considerations

We propose that isohemagglutinins comprise distinct clones, each defined by ABO specificity, with additional reactivity toward unrelated structures such as pneumococcal CWP and specific CPSs. Although we did not directly investigate the mechanisms behind the polyreactivity of individual clones, previous studies suggest that paratope plasticity, structural isomerism, and positional flexibility of epitope binding may contribute to this phenomenon (see [2, 4] for review). Structural mimicry between ABO antigens and bacterial glycoconjugates may also play a role.

Although we affinity-purified IgG isohemagglutinins and confirmed their reactivity with pneumococci, our data do not elucidate the original immunogens responsible for eliciting these antibodies. In principle, each clone’s reactivity with ABO antigens and/or pneumococcal polysaccharides could represent incidental reactivity arising by chance in individuals lacking strict immunological tolerance to these structures.

### Comparison with existing literature

Isohemagglutinins have long been known to bind pneumococci, though earlier observations were often attributed to contamination with ABO-like structures in early vaccine preparations [34–37]. However, modern vaccine-grade CPS are produced without (and tested for) such contaminants [38]. The nature of the previous contaminants is unclear, but based on our findings, we suspect that CWP was at least in part responsible. CWP is present in pneumococcal vaccines [39] but in variable amounts across CPS preparations [32] and production procedures [40]. Although the CWP content is reduced in modern pneumococcal vaccines, anti-CWP antibodies rise after vaccination [41]. Notably, this increase is not typically reflected in rising isohemagglutinin titers [42], possibly because CWP-reactive isohemaglutinins represent only a minor fraction of total isohemagglutinins (analogous to the situation for anti-αGal antibodies [14]). Slight increases in isohemagglutinin levels were indeed detected after pneumococcal vaccination when assayed with a more sensitive technique (surface plasmon resonance) [42].

Previous studies examining the association between ABO blood group and pneumococcal disease risk have produced inconsistent findings. Reed et al. [43] suggest a potential protective effect of anti-A antibodies, noting that blood group B individuals are markedly underrepresented among pneumococcal cases in Albuquerque, New Mexico; but not in nearby Gallup County, where group B was rare at the time due to a predominantly Native American population. However, the small sample size (*n* = 88) and lack of adjustment for ethnic variation in ABO distribution leaves the study prone to confounding by population structure and social determinants of health. The observed associations may therefore reflect demographic imbalances rather than a true biological effect. Others have approached the question differently by investigating direct interactions between pneumococci and ABO antigens; suggesting that such binding could influence susceptibility across blood groups [44–46]. Our population-based analysis, which included thousands of individuals, found no broad association between ABO type and IPD risk, aside from a modest inverse association for serotype 9V and inferred anti-A isohemagglutinins. These findings strongly suggest that neither ABO type nor isohemagglutinins have a major impact on susceptibility to IPD.

### Clinical and immunological relevance

We focused on isohemagglutinins of the IgG class due to their prevalence in our preparations, their high relative purity, and the known protective role of IgG antibodies against pneumococcal infections [11, 12]. We cannot exclude that dimeric IgA and especially IgM isohemagglutinins, which have greater potential for multivalent binding, may exhibit broader reactivity; however, this was not investigated in the present study.

Importantly, the observed isohemagglutinin reactivity did not translate into a strong association between ABO blood type and the risk of IPD at the population level. One explanation may be that much of the antibody reactivity was directed against the common CWP, and anti-CWP antibodies are widely regarded as non-protective in humans [47–49]. However, some isohemagglutinins displayed serotype reactivity, and such antibodies are generally considered protective [11, 12]. In this context, isohemagglutinin binding might not result in Fc conformations that support downstream effector functions, as suggested for anti-αGal antibodies on selected bacteria [50, 51]. Furthermore, the composition and abundance of polyreactive isohemagglutinins is likely to vary between individuals, which could limit any population-wide protective effect. Moreover, an isohemagglutinin reactive to a given serotype may represent an insignificant fraction of an individual’s total antibodies against that serotype. Additionally, in individuals unable to produce pneumococcal-reactive isohemagglutinins due to expression of the corresponding ABO antigen, other antibodies may simply compensate.

The lack of a strong association may not be surprising from an evolutionary perspective, given the persistence of all ABO types in the human population. If certain blood groups conferred significant protection against ubiquitous pathogens like pneumococci, we would expect natural selection to reduce the frequency of the more susceptible ABO types. This is consistent with the generally weak or inconsistent associations between ABO type and, by extension, isohemagglutinin production and infection risk in broader contexts [52–54].

### Study limitations

Our study has several limitations. The use of pooled plasma limits assessment of individual variability in isohemagglutinin repertoires. The large pool was chosen to generate sufficient antibody yields for purification and to provide an approximate measure of average human isohemagglutinin reactivity. While this approach captures broad trends, it does not reflect potential donor-specific differences in binding profiles. Also, only IgG reactivity was studied, which may underestimate total antibody binding. Functional assays such as opsonophagocytic killing were not done, precluding conclusions about protective efficacy. Finally, flow cytometry identified “bulk” bacterial binding, but not precise epitopes.

### Future directions

Future work should isolate individual isohemagglutinin clones to clarify their specificities and functional roles. Examining isohemagglutinins at the level of individual donors will also be important to assess potential variability in binding patterns and to determine whether distinct reactivity profiles exist among humans. Structural studies could reveal how certain clones achieve dual recognition of ABO and microbial antigens. Extending these approaches to other naturally occuring antibodies, including those targeting protein antigens, may uncover shared mechanisms of synergistic polyreactivity and broaden the understanding of innate humoral immunity.

## Conclusion

Our study demonstrates that isohemagglutinins, previously thought to be narrowly restricted to ABO antigens, exhibit synergistic polyreactivity toward pneumococcal polysaccharide antigens. This reactivity involves both common and serotype-specific antigens, revealing a broader functional repertoire for antigen-defined antibodies than previously recognized. Despite their broad-spectrum pneumococcal reactivity, our epidemiological analyses suggests that isohemagglutinins do not provide significant protection against IPD. These insights not only reshape the functional landscape of naturally occurring antibodies but also underscore the complexity of their contribution to host defense.

## Supplementary Information

Below is the link to the electronic supplementary material.

Supplementary Material 1

## Data Availability

Data supporting the findings of this study are available from the corresponding author upon reasonable request.
