# Supplementary material for: Isohemagglutinins exhibit synergistic polyreactivity toward Streptococcus pneumoniae surface antigens: implications for broad-spectrum reactivity of human antibodies
Source: Med Microbiol Immunol. 2026 Jan 9;215(1):5. doi: 10.1007/s00430-025-00862-y (PMC12789223; doi:10.1007/s00430-025-00862-y)
Supplement: Supplementary file 1 — Supplementary Material 1 [file 430_2025_862_MOESM1_ESM.pdf]

Supplementary Information

for:

”Isohemagglutinins Exhibit Synergistic Polyreactivity Toward *Streptococcus pneumoniae* Surface Antigens: Implications for Broad-Spectrum Reactivity of Human Antibodies”

Authors

Jens Magnus Bernth Jensen[1,2], Ole Schmeltz Søgaard[3], Mikkel Steen Petersen[1], Steen Hoffmann[4], Bjarne Kuno Møller[1], Uffe B. Skov Sørensen[5], and Steffen Thiel[5]

Author affiliations

[1]Department of Clinical Immunology, Aarhus University Hospital, Denmark
[2]Department of Molecular Medicine, Aarhus University Hospital, Denmark
[3]Department of Infectious Diseases, Aarhus University Hospital, Denmark
[4]Department of Bacteria, Parasites and Fungi, Statens Serum Institut, Denmark
[5]Department of Biomedicine, Aarhus University, Denmark

Corresponding author

Jens Magnus Bernth Jensen
Department of Clinical Immunology and Department of Molecular Medicine, Aarhus University Hospital, Denmark
Email: jmbj@clin.au.dk

Statement on Content

This supplementary file contains additional figures, tables, and methods that support the findings presented in the main manuscript.

Table of Contents

Fig S1 Western Blot analysis of isohemagglutinin preparations 3

Fig. S2 Inhibition of antibody binding to ABO-antigen-coated surfaces by RBCs 4

Fig. S3 Antibody binding to blood group A antigen as a function of relative antibody concentration 5

Fig. S4 Antibody binding to blood group B antigen as a function of relative antibody concentration 5

Fig. S5 Antibody levels in αB-IH compared to the plasma pool used for affinity purification....6

Fig. S6 Recovery of selected antibodies in fractions from affinity purification procedures 7

Table S1 Frequency of Invasive Pneumococcal Disease by Serotype in Danish Patients (1966–2014) 8

Fig. S7 Dilution series of nhIgG on an *S. pneumonia* strain 9

Fig. S8 Inhibition of αA-IH reactivity to pneumococcal strains by soluble pneumococcal polysaccharides 10

Fig. S9 Inhibition of isohemagglutinin reactivity to pneumococcal strains by soluble CWP....11

Fig. S10 Percentage of IgG binding signal remaining after inhibition with soluble CWP 12

Fig. S11 ORs for IPD by ABO blood group, stratified by pneumococcal serotype. 13

Supplementary Data for:” **Isohemagglutinins Exhibit Synergistic Polyreactivity Toward *Streptococcus pneumoniae* Surface Antigens: Implications for Broad-Spectrum Reactivity of Human Antibodies**”

Fig S1 Western Blot analysis of isohemagglutinin preparations

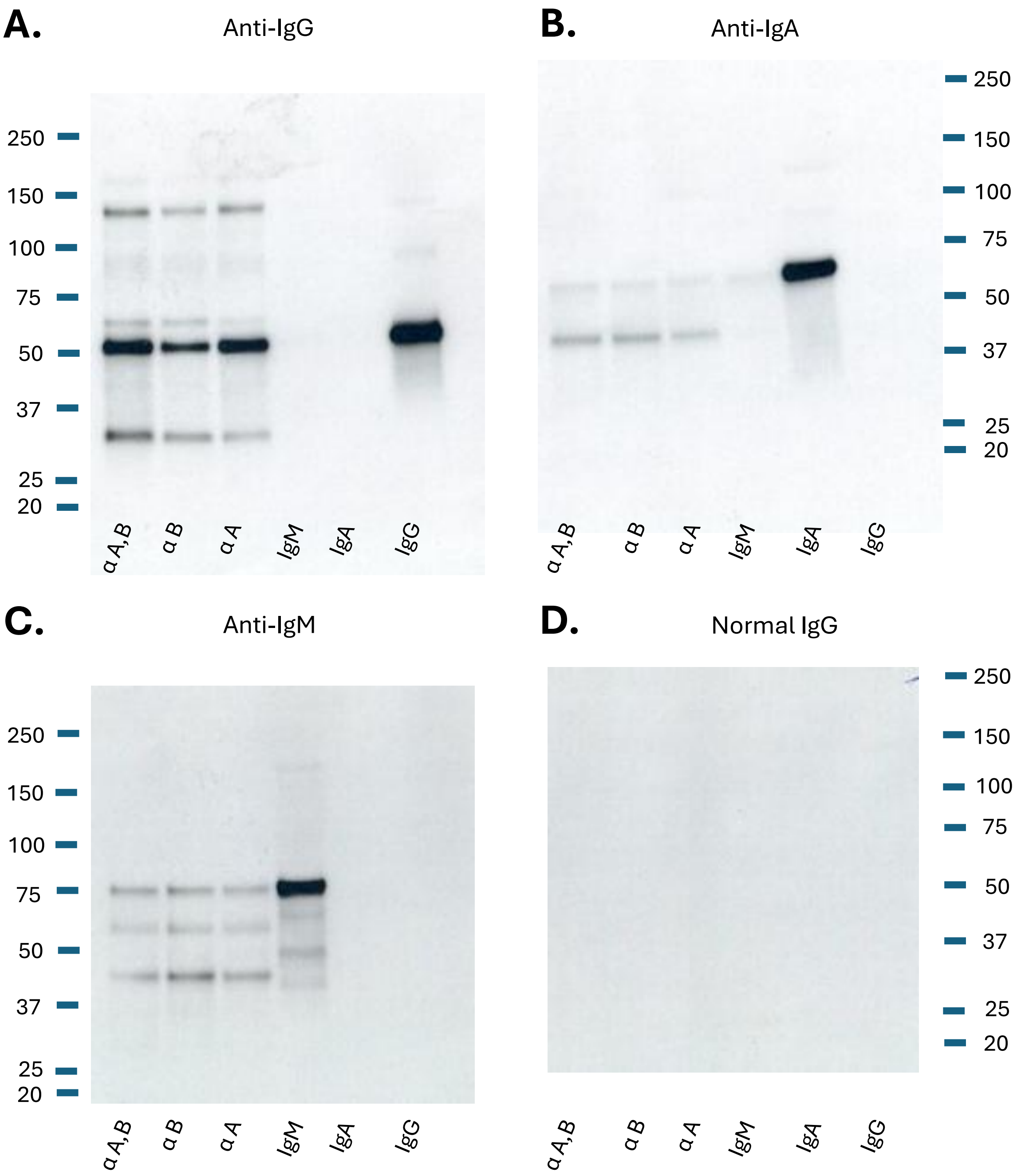

Fig S1 The wells were loaded with αA,B-IH (0.5 μg), αB-IH (1 μg), αA-IH (0.5 μg), IgG (0.2 μg), IgA (0.2 μg), and IgM (0.2 μg). Blots were developed using rabbit anti-human IgG (**A**), IgA (**B**), IgM (**C**), and normal rabbit immunoglobulin (**D**).

Fig. S2 Inhibition of antibody binding to ABO-antigen-coated surfaces by RBCs

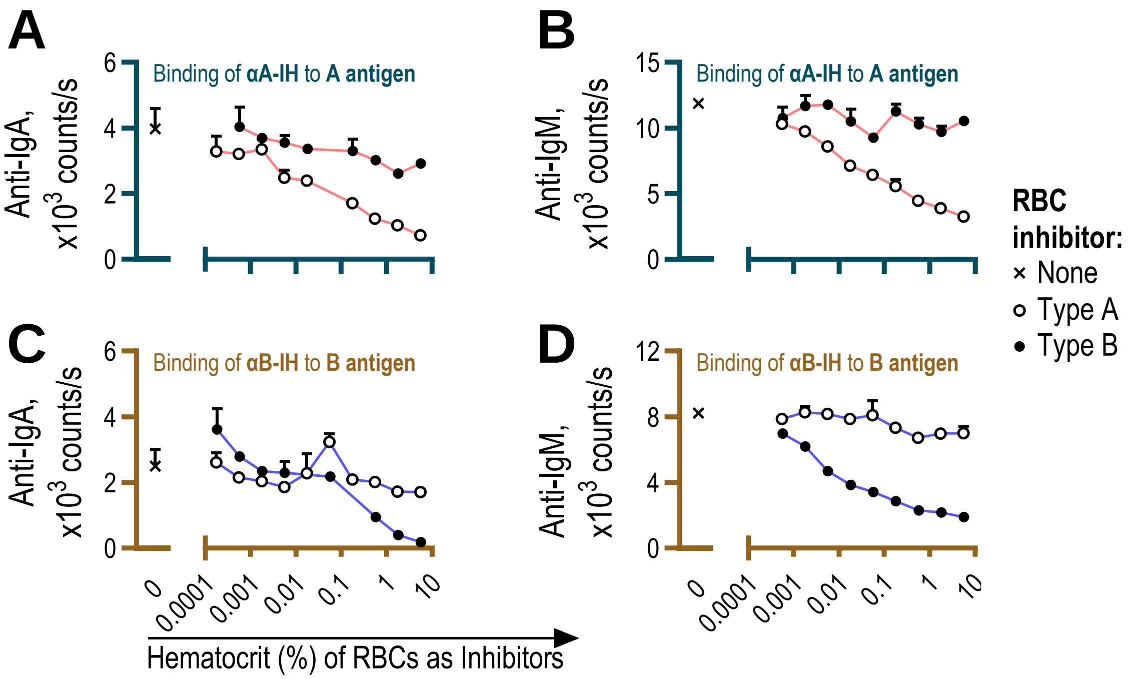

The αA-IH preparation (1:10,000 dilution in sample buffer) was incubated with type A or B RBCs at varying hematocrit levels and tested for IgA (**A**) and IgM (**B**) binding to an A-antigen-coated surface. The αB-IH preparation (1:7,500 dilution) was tested similarly on a B-antigen-coated surface, with IgA antibodies in panel **C** and IgM antibodies in panel **D**. Data are presented as mean binding signals for each antibody class, with the sample buffer-only signal subtracted, and standard deviations from duplicate experiments, plotted as a function of RBC hematocrit. Data points with a coefficient of variation above 15% in duplicate experiments were omitted.

Fig. S3 Antibody binding to blood group A antigen as a function of relative antibody concentration

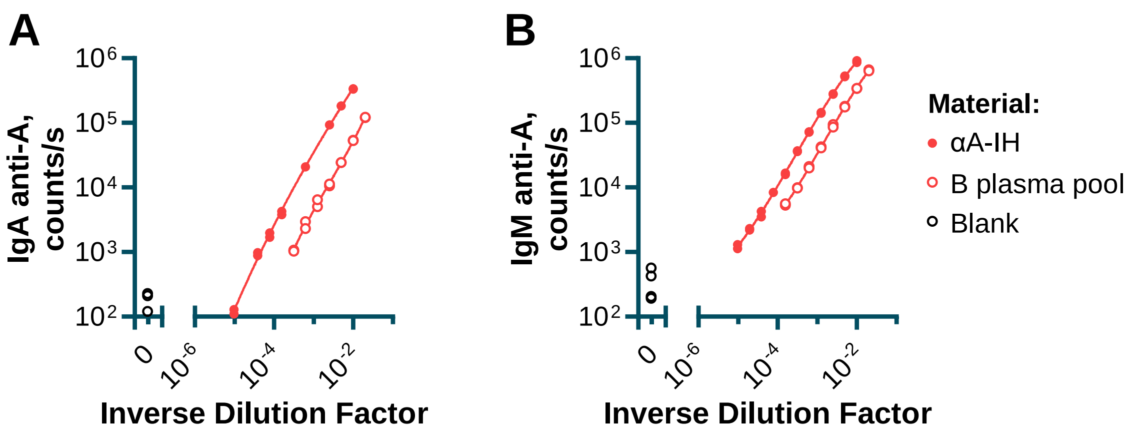

Binding of IgA (**A**) and IgM antibodies (**B**) were measured in two dilution series: one from affinity-isolated αA-IH and another from its starting material (ABO type B plasma pool diluted in TBS/Tw). The x-axis shows the inverse dilution factor on a $\log_{10}$ scale, representing relative antibody concentration. The parallel curves in each of the two panels indicate similar avidity of anti-A antibodies in both preparations.

Fig. S4 Antibody binding to blood group B antigen as a function of relative antibody concentration

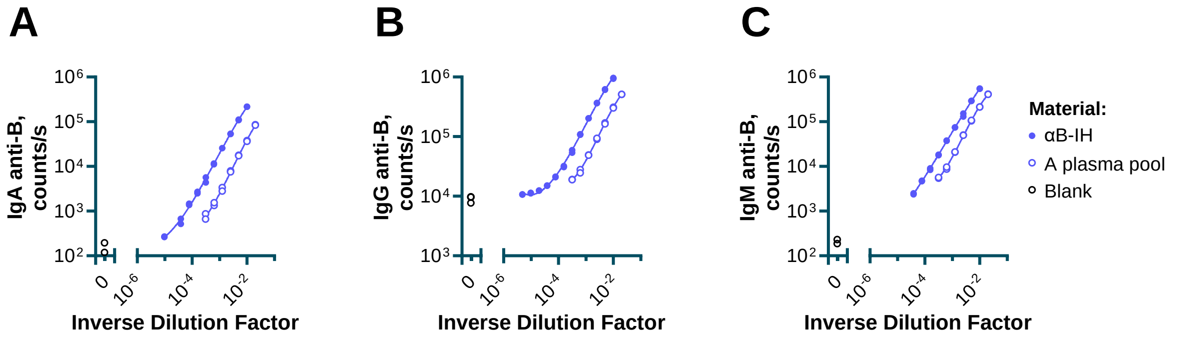

Binding of IgA (A), IgG (B), and IgM antibodies (C) were measured in two dilution series: one from affinity-isolated αB-IH and another from its starting material (ABO type A plasma pool diluted in TBS/Tw). The x-axis shows the inverse dilution factor on a $\log_{10}$ scale, representing relative antibody concentration. The parallel curves in each of the three panels indicate similar avidity of anti-B antibodies in both preparations.

Fig. S5 Antibody levels in αB-IH compared to the plasma pool used for affinity purification

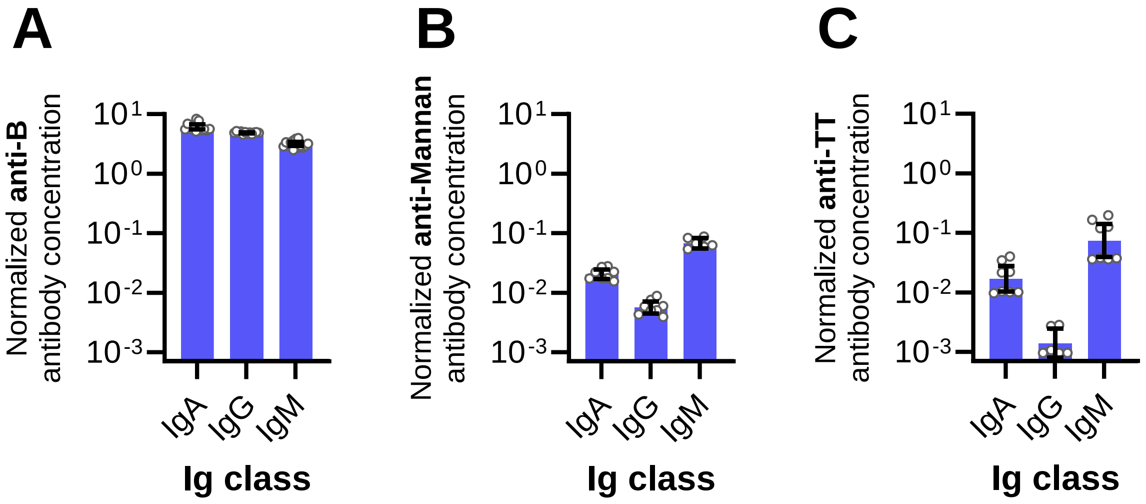

Histograms showing anti-B (A), anti-mannan (B), and anti-TT (C) antibodies in αB-IH across different immunoglobulin classes. Antibody levels are normalized to their respective concentrations in the ABO type A plasma pool used for affinity purification of αB-IH. Samples were analyzed by TRIFMA, with bars representing the mean of repeated measurements and error bars indicating 95% confidence intervals.

Fig. S6 Recovery of selected antibodies in fractions from affinity purification procedures

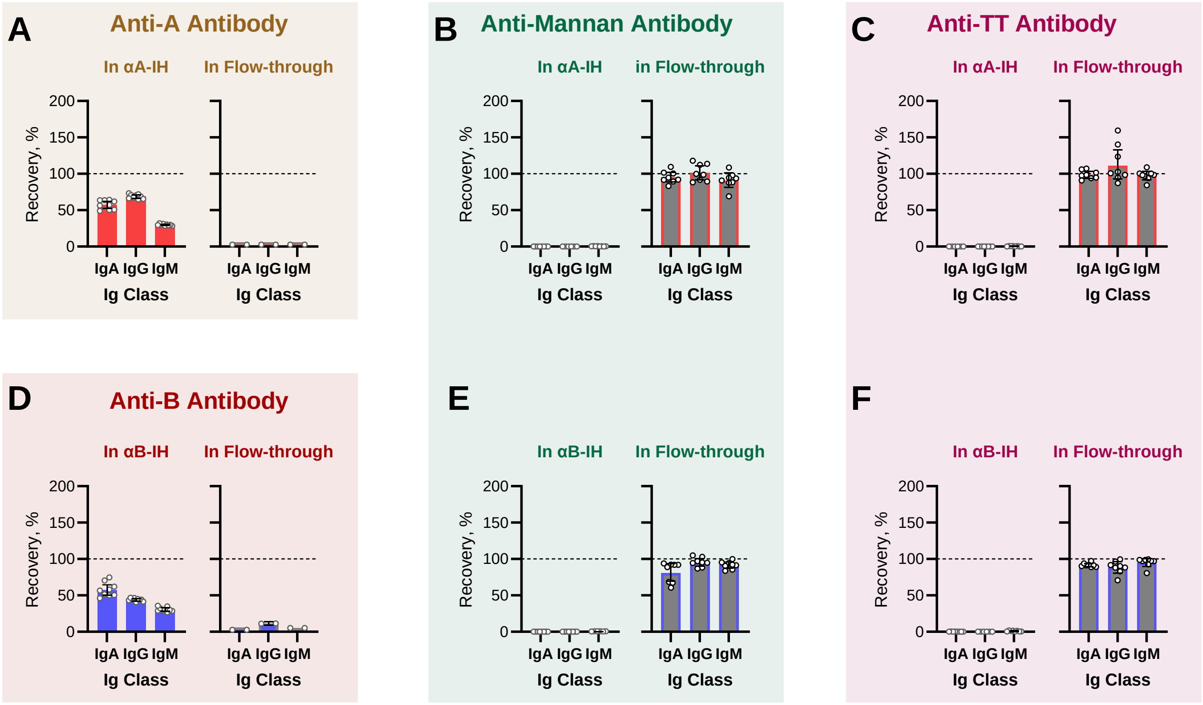

The recovery of different antibodies in αA-IH and αB-IH fractions, as well as their corresponding flow-throughs, was assessed relative to their original amounts in the plasma pools before affinity purification. Antibody concentrations were quantified using time-resolved immunofluorometric assays (TRIFMAs) for IgA, IgG, and IgM against blood group A antigen, blood group B antigen, mannan, and TT in each fraction. Antibodies against mannan and TT were measured as controls to assess nonspecific binding or contamination. The total amount of each antibody in each fraction was determined by multiplying its concentration by the respective fraction volume, allowing calculation of recovery percentages. The top panels show antibody recovery in αA-IH and its flow-through, while the bottom panels display recovery in αB-IH and its flow-through. Bars represent mean values from repeated measurements, and error bars indicate 95% confidence intervals.

The anti-A and anti-B isohemagglutinins, which were not recovered in αA-IH and αB-IH or their corresponding flow-throughs, were most likely retained in the columns.

Table S1 Frequency of Invasive Pneumococcal Disease by Serotype in Danish Patients (1966–2014)

**Serotype**	**Cases**	**Proportion (%)**	**Cumulative Proportion (%)**
1	1,956	13.3	13.3
7F	1,096	7.45	20.8
14	1,034	7.03	27.8
4	1,011	6.88	34.7
3	828	5.63	40.3
8	779	5.30	45.6
9V	714	4.86	50.4
12F	679	4.62	55.1
23F	539	3.67	58.7
22F	507	3.45	62.2
19F	481	3.27	65.5
9N	477	3.24	68.7
6B	466	3.17	71.9
19A	451	3.07	74.9
6A	369	2.51	77.4
18C	296	2.01	79.5
11A	267	1.82	81.3
33F	218	1.48	82.8
20	210	1.43	84.2
24F	205	1.39	85.6
38	165	1.12	86.7
10A	155	1.05	87.8
23A	140	0.952	88.7
35F	138	0.939	89.6
5	128	0.871	90.5
16F	127	0.864	91.4
15A	122	0.830	92.2
15B	113	0.768	93.0
6C	111	0.755	93.7
15C	91	0.619	94.3
'Rough'	139	0.945	95.3
Others	692	4.71	100
**Total**	14704	100	—

Data from the Danish national surveillance registry for invasive pneumococcal disease. 'Rough' refers to unencapsulated pneumococci. 'Others' include various less common serotypes and strains with uncertain serotype designation.

Fig. S7 Dilution series of nhIgG on an *S. pneumonia* strain

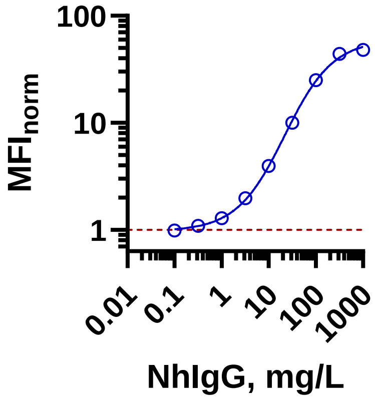

Exemplification of the relationship between nhIgG concentration and the derived binding signal for IgG bound to the serotype 9V strain. The experiment was conducted as described in the main text and analyzed on a NovoCyte Quanteon flow cytometer, which has higher sensitivity compared to the NovoCyte 3000 used for other analyses. This increased sensitivity improves confidence in negative results.

Fig. S8 Inhibition of αA-IH reactivity to pneumococcal strains by soluble pneumococcal polysaccharides

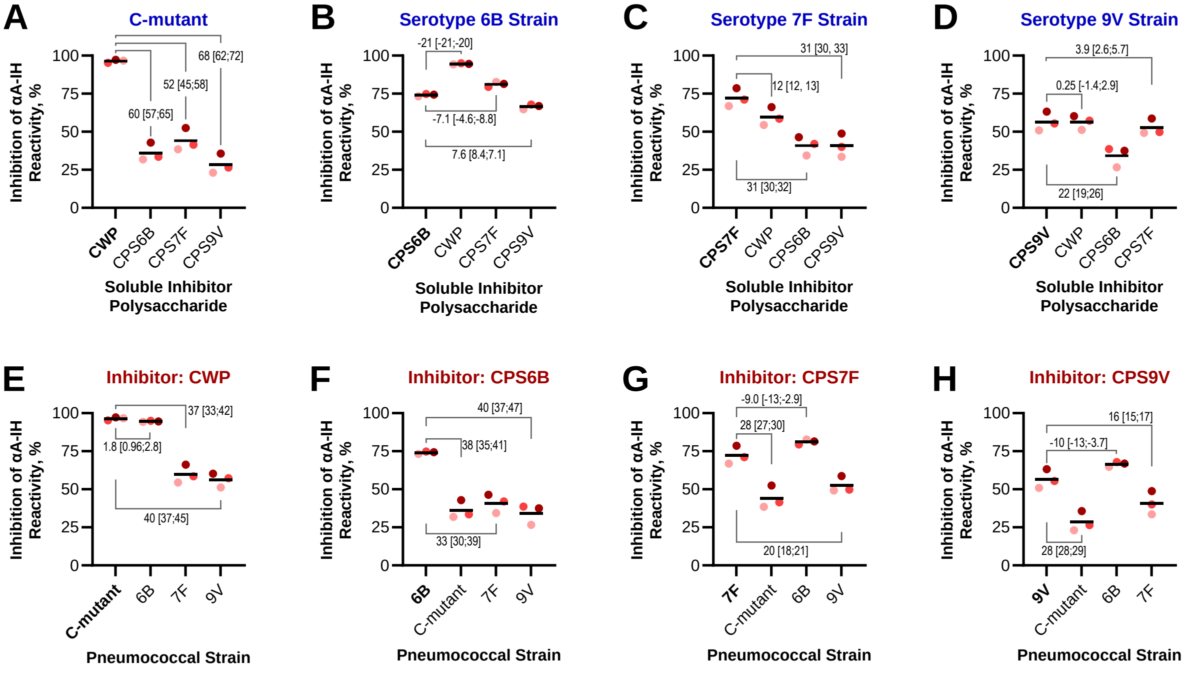

Data presented in **Fig. 5** of the main text, further stratified by pneumococcal strain (top row) and soluble inhibitor polysaccharide (bottom row). The data points from experiments conducted on the same day are shaded in similar red tones. The average difference in inhibition relative to that achieved with homologous CPS was calculated using a paired bootstrapping approach to estimate the 95% CI.

Fig. S9 Inhibition of isohemagglutinin reactivity to pneumococcal strains by soluble CWP

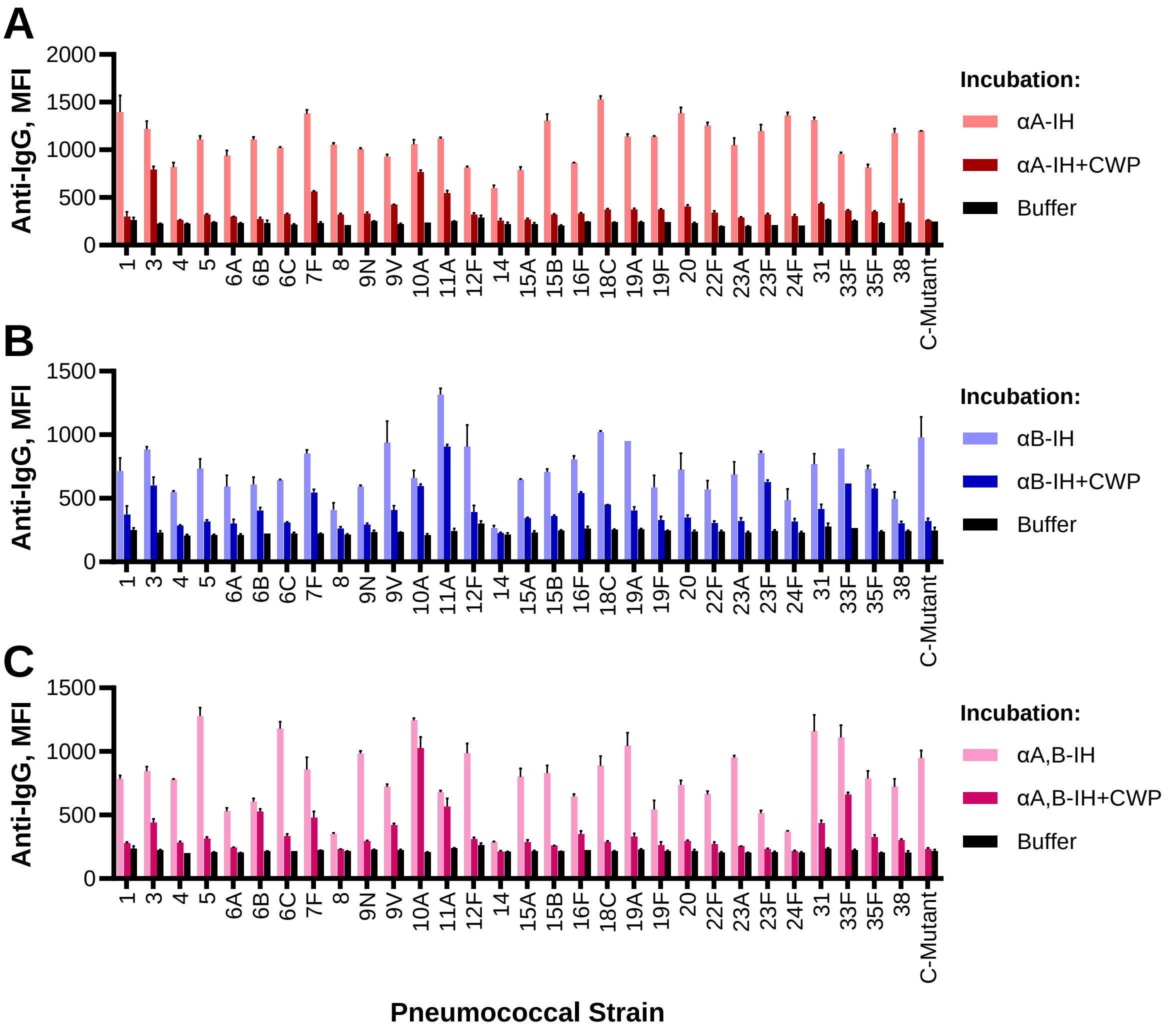

Isohemagglutinin preparations or buffer-only controls were incubated with pneumococcal strains in the presence or absence of soluble CWP. Isohemagglutinin dilutions were optimized in preliminary experiments to yield an MFI_{norm} of ≤5 for each strain, ensuring a sufficient dynamic range for detecting inhibition. The binding of αA-IH and αA,B-IH was challenged with CWP at 0.1 g/L, whereas αB-IH was challenged with 1 g/L to account for its higher apparent avidity for CWP on cells. Bound IgG was detected using fluorescently labeled $F(ab')_2$ anti-human IgG and analyzed by flow cytometry. Bars represent the mean of two independent experiments, with error bars indicating standard deviation.

Fig. S10 Percentage of IgG binding signal remaining after inhibition with soluble CWP

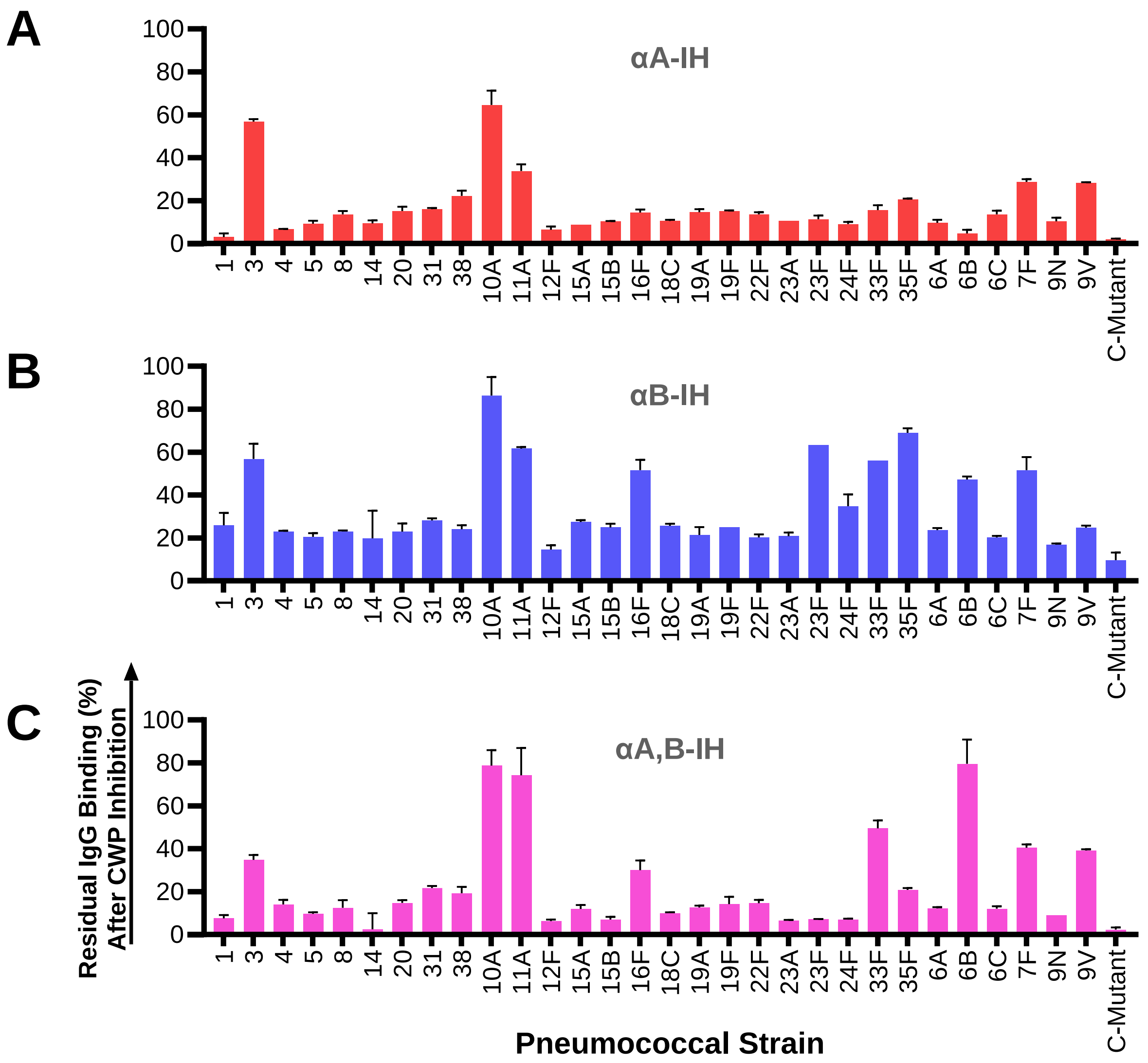

Percentage of IgG binding signal remaining after inhibition with soluble CWP. Data correspond to the previously presented supplementary figure but are expressed as the IgG binding signal over background following CWP inhibition ($MFI_{IH+CWP} - MFI_{Buffer}$) as a percentage of the IgG binding signal over background without CWP inhibition ($MFI_{IH} - MFI_{Buffer}$) for each pneumococcal strain. As a control for the efficiency of CWP adsorption, binding to the C-mutant strain was abolished for αA-IH and αA,B-IH, whereas only minimal residual reactivity (10%) persisted for αB-IH.

Fig. S11 ORs for IPD by ABO blood group, stratified by pneumococcal serotype.

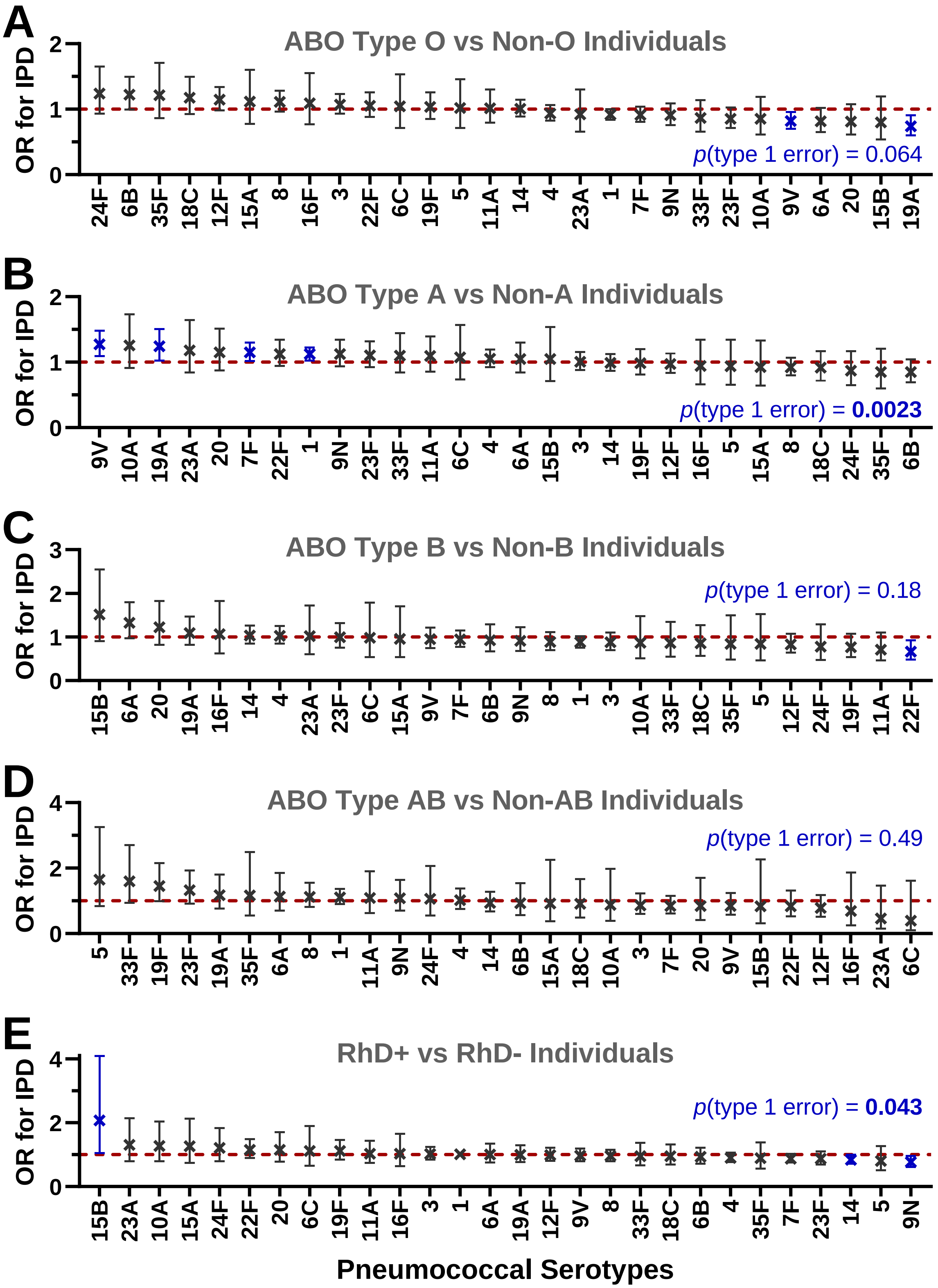

Nationwide Danish registry data were analyzed, including all available ABO and RhD blood type records and IPD cases in individuals aged ≥2 years. Only serotypes with at least 100 cases were included. For each panel, ORs were calculated as: (number of IPD cases in individuals with the indicated blood group / total number of individuals with that blood group) divided by (number of IPD cases in individuals without the indicated blood group / total number of individuals without that blood group). Comparisons shown are: ABO type O vs non-O (**A**), type A vs non-A (**B**), type B vs non-B (**C**), type AB vs non-AB (**D**), and RhD-positive vs RhD-negative individuals (**E**). The RhD comparison serves as a control, as RhD antigens do not elicit naturally occurring antibodies. Error bars represent 95% CIs calculated using the Baptista-Pike method. A horizontal dotted line at OR = 1 indicates no difference in odds between groups.

None of the ORs remained statistically significant after corrections for multiple testing using the Bonferroni method. To assess whether the observed number of confidence intervals not spanning 1 could be attributed to chance (type I error), we calculated the probability of observing this number using the multinomial probability formula:

$$P(\text{type 1 error}) = \frac{n!}{k_{\text{below}}!\,k_{\text{above}}!\,(n - k_{\text{below}} - k_{\text{above}})!} \times p^{n-k_{\text{below}}-k_{\text{above}}} \times q_{\text{below}}^{k_{\text{below}}} \times q_{\text{above}}^{k_{\text{above}}}$$

where n is the total number of confidence intervals, p is the probability a confidence interval spans 1, and q_{below} and q_{above} are the probabilities of a confidence interval excluding 1 below or above, respectively. This analysis considers only whether CIs span 1—not the magnitude of the deviation.

Among the ABO group comparisons, only individuals with blood group A showed a statistically significant enrichment of non-overlapping CIs after correction for multiple testing, with a type I error probability of p = 0.0023. Notably, all four serotypes contributing to this signal (9V, 19A, 7F, and 1) had ORs with CIs entirely above 1, indicating a consistent directionality of increased risk. In contrast, a similarly low p-value was observed for the RhD comparison (p = 0.043), despite RhD antigens not being associated with naturally occurring antibodies and thus serving as a negative control. However, in the RhD analysis, the directions of effect varied (one CI was above 1 and two were below) suggesting these deviations are more likely attributable to random variation. Together, these findings imply that the signal observed for blood group A may reflect a weak but non-random association with increased susceptibility to IPD from select pneumococcal serotypes. Nonetheless, given the borderline nature of these results and the presence of nominal associations in the RhD control, these findings should be interpreted with caution and warrant validation in independent cohorts.
